# Human IDO-competent, long-lived immunoregulatory dendritic cells induced by intracellular pathogen, and their fate in humanized mice

**DOI:** 10.1038/srep41083

**Published:** 2017-02-15

**Authors:** Rajeev K. Tyagi, Brodie Miles, Rajesh Parmar, Neeraj K. Garg, Sarat K. Dalai, Babak Baban, Christopher W. Cutler

**Affiliations:** 1 Department of Periodontics, College of Dental Medicine, Georgia Regents University, Augusta, GA 30912, USA; 2 Division of Infectious Diseases, Anschutz Medical Campus, University of Colorado Denver, Aurora, CO 80045, USA; 3 Institute of Science, Nirma University, Sarkhej-Gandhinagar Highway, Ahmedabad 382481, Gujarat, India; 4 Drug Delivery Research Group, University Institute of Pharmaceutical Sciences, UGC center of Advanced Studies, Panjab University, Chandigarh, India; 5 Department of Oral Biology, Georgia Regents University, Augusta, GA 30912, USA

## Abstract

Targeting of myeloid-dendritic cell receptor DC-SIGN by numerous chronic infectious agents, including *Porphyromonas gingivalis*, is shown to drive-differentiation of monocytes into dysfunctional mDCs. These mDCs exhibit alterations of their fine-tuned homeostatic function and contribute to dysregulated immune-responses. Here, we utilize *P. gingivalis* mutant strains to show that pathogen-differentiated mDCs from primary human-monocytes display anti-apoptotic profile, exhibited by elevated phosphorylated-Foxo1, phosphorylated-Akt1, and decreased Bim-expression. This results in an overall inhibition of DC-apoptosis. Direct stimulation of complex component CD40 on DCs leads to activation of Akt1, suggesting CD40 involvement in anti-apoptotic effects observed. Further, these DCs drove dampened CD8^+^ T-cell and Th1/Th17 effector-responses while inducing CD25^+^Foxp3^+^CD127^−^ Tregs. *In vitro* Treg induction was mediated by DC expression of indoleamine 2,3-dioxygenase, and was confirmed in IDO-KO mouse model. Pathogen-infected & CMFDA-labeled MoDCs long-lasting survival was confirmed in a huMoDC reconstituted humanized mice. In conclusion, our data implicate PDDCs as an important target for resolution of chronic infection.

Dendritic cells (DC), major sentinels of immune system, are involved in sensing of foreign antigens, and subsequent antigen processing and presentation to lymphocytes. DCs are main antigen-presenting cells (APC) of immune system and are connecting link between adaptive and non-adaptive immune responses. The functional diversity and generation of adaptive immunity by DCs is crucial to study pathogenesis of diseases caused by infectious agents, vaccine responses, cancers, and autoimmune diseases^1^,^2^,^3^,^4^,^5^. The conventional mode of differentiation of CD14^+^ monocytes into immature monocyte-derived DCs (MoDCs) can be induced by IL-4 and GM-CSF *in vitro*. We have recently identified a novel, pathophysiological non-canonical pathway of DC differentiation from CD14^+^ progenitors by bacteremic pathogen *Porphyromonas gingivalis*^6^. Blood mDCs express low levels of pattern recognition receptor (PRR) DC-SIGN, and dermal DCs have high levels of DC-SIGN, a key receptor involved in uptake of chronic pathogen, *P. gingivalis* through its minor (Mfa-1) fimbriae. The chronic periodontitis patients show an increase in DC-SIGN^+^ CD1c^+^ mDCs in peripheral blood^7^,^8^. These mDCs are carriers or host for *P. gingivalis*^9^, and disseminate to rupture prone atherosclerotic plaques *in situ*^8^. Further, MoDCs and mDCs infected via DC-SIGN ligation were shown to have inhibited apoptosis through annexin V staining *in vitro*^6^.

Apoptosis is involved in cellular development, elimination of damaged cells, and establishment of cell homeostasis^10^,^11^,^12^. The activation of phosphatidyl inositol kinase and its downstream effecter kinase, Akt1 inhibits apoptosis^10^. Further, downstream effectors of Akt1, Bcl-2 family^10^,^11^, are anti-apoptotic, and thus help to extend cell survival^10^,^12^. The DC-specific C-type lectin DC-SIGN has been established as a key immunomodulator in the induction of immune responses for many pathogens, including *P. gingivalis*^13^, *Mycobacterium tuberculosis*^14^, and *Helicobacter pylori*^15^. Interestingly, DC-SIGN ligation leads to downstream Akt1 activation^16^ which in turn phosphorylates and inactivates Forkhead box class O (FOXO) family of transcription factors. Upon phosphorylation by Akt1, FOXO proteins are excluded from the nucleus, and becomes transcriptionally inactive, while shuttling from nucleus to the cytoplasm leads to their sequestration or degradation^17^,^18^,^19^. FOXO inactivation contributes to extended cell survival as FOXO members are capable of activating genes encoding pro-apoptotic molecules including Bcl-2 family member, Bim.

The immunopathogenesis of chronic periodontitis (CP) and other chronic inflammatory diseases^20^,^21^,^22^, has been linked with Th2 and Treg immune biases. The DC-SIGN targeting by *P. gingivalis* Mfa-1 fimbriae elicits Th2 biased response in monocyte-derived DCs (MoDCs)^23^. The role of DC-SIGN targeting in the production of indoleamine-2,3-dioxygenase (IDO), and its contribution for the modulation of immune system and induction of Treg response is not clear. However, IDO has been established as a crucial player in determining Treg function and maintenance (Nair *et al*., 2004; Royer *et al*., 2010). IDO helps create a tolerogenic state and induces immunosuppression both by direct suppression of T cells and enhancing the production of local Tregs^24^,^25^. The induction of Tregs through DC activity has been shown to promote disease progression^12^,^17^ and to mediate pathogen immune escape^11^. Present study supports our hypothesis that IDO is a crucial determinant in inducing Tregs by the modulation of inflammatory responses in IDO-KO mouse model of gingivitis and its role in limiting inflammatory responses in pathophysiological settings such as periodontitis.

Current study investigated the ability of pathogen differentiated dendritic cells (PDDCs) to contribute to persistent *P. gingivalis* infection and chronic inflammation, through inhibition of PDDC apoptosis and their alteration of effector responses, respectively.

To address the role of fimbriae in this regard we utilized defined bacterial mutants, that solely express minor fimbriae (Mfa-1^+^Pg), major fimbriae (FimA^+^Pg) or are deficient in both fimbriae (MFB) (Table 1). We utilized isogenic mutant strains of *P. gingivalis* that express different fimbrial adhesins (Table 1) and observed that PDDCs generated by strains expressing Mfa-1 fimbriae exhibited activation of Akt1 and inactivation of FOXO1. The inhibition of Akt1 partially prevented anti-apoptotic effects of Mfa-1/DC-SIGN interaction. Our study further shows that these long-lived PDDCs were unable to activate CD8^+^ or Th1/Th17 effector responses critical to pathogen elimination, but rather induced a robust Treg response.

As *P. gingivalis* reportedly induced chemokine paralysis, inhibits IL-12 production, and suppresses complement activation which rescues it from host immunity^26^,^27^, we decided to track *P. gingivalis* loaded MoDCs in huMoDC reconstituted humanized NSG (NOD/SCID IL2Rg^−/−^). The humanized mouse was prepared by ameliorating residual non-adaptive immune response by the treatment of clodronate-loaded liposome^28^,^29^, and as others^30^,^31^,^32^,^33^, we saw sizeable human cell grafting reconstituted humanized mice. Our results suggest that DC-SIGN expressing *P. gingivalis* strains (WT & Mfa-1) show inhibited apoptosis and thereby confer extended survival on pathogen. we decided to track CMFDA labeled and *P. gingivalis* loaded MoDCs. Therefore, we recorded signals via whole body imaging on live animals emitted from CMFDA labeled monocytes (MN) and MoDCs lasting for more than 10 days in deep-seated organs. This observation supports our *in vitro* findings showing the long-lived DCs when pulsed with DC-SIGN expressing *P. gingivalis.* We hardly saw bacteria pulsed DCs circulating in the periphery of huMoDCs reconstituted humanized mouse 48 hr post-administration. However, signals emitted from CMFDA labelled MoDCs were recorded in deep-seated organs until day 10 post-injection. Furthermore, results obtained from immunofluorescence assay carried out on tissue sections were suggestive of sequestration like mechanisms employed by bacteria to escape host’s immunity and thereby reside longer in the heart. In conclusion, we hypothesize that pathogen-DC complex might operate as a molecular transducer of signals in inhibiting apoptosis, and IDO-dependent induction of local regulatory T cells playing a crucial role in immunosuppression and establishment of immune homeostasis.

## Results

### Transcriptome analysis shows pathogen differentiated DCs are distinct from monocytes and monocyte-derived DC

As our group recently discovered and validated generation of non-canonical DCs differentiated by *P. gingivalis,* we obliged to characterize their gene expression profile by customized PCR micro-array (Table 2, Supplementary Fig. S2). The fundamental cytokines, chemokines, and transcription factors playing an instrumental role in DC biology were analyzed on PDDC generated by the isogenic mutant(s) of *P. gingivalis*. Monocyte-derived DC (MoDC) and PDDC expression patterns were normalized to baseline monocyte expression. PDDC were seen in the immature state confirmed by the expression of various accessory markers (CD86 & CD40). In addition, PDDCs showed enhanced expression of pro-inflammatory cytokine, IL-6 and anti-inflammatory cytokine, IL-10 as well as sizeable increases of regulatory markers, IDO and TGFBR2.The higher expression of ADORA2B induces anti-inflammatory responses in DPG-PDDC compared with MoDC and DCs derived from other isogenic mutants (WT, MFI & MFB-DC). We observed markedly higher expression of survivin (BIRC5) and FOXO1 (Table 2, Supplementary Fig. S2) by minor fimbriae-differentiated DC (DPG-DC), which in turn indicated inhibited apoptosis (suppressed pro-apoptotic Bim/Bcl2-l1) occurring in PDDCs compared to that with MoDCs.

### Pathogen-derived DCs are anti-apoptotic

**Figure 1:**
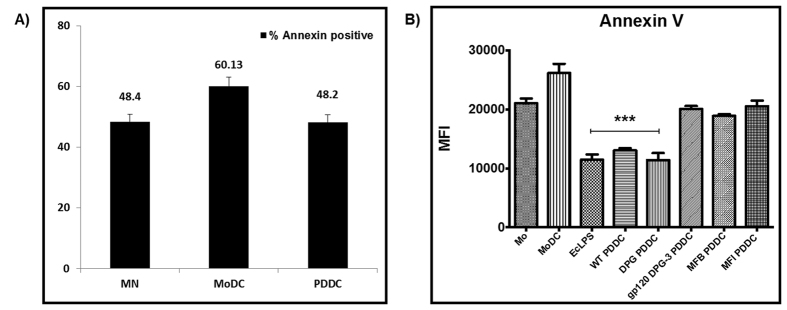
MoDCs show higher rate of apoptosis than PDDCs. (**A**) Unpulsed MoDC and Pg-wt differentiated DC (WT-PDDC) were stained with propidium iodide staining solution using apoptosis detection kit (Annexin V staining kit, eBioscience). Cells were washed in RPMI supplemented with 10% FBS, and cytospin in order to perform immunofluorescence assay. Apoptotic MoDC and PDDC (arrows), with a characteristic condensed, fragmented, brighter nucleus than non-apoptotic DCs. Insets, view of PI staining of representative apoptotic cells. Scale bars: DCs, 35 μm; Monocytes (MN), 15 μm, (**B**) Unpulsed MoDC and Pg-wt differentiated DC (WT-PDDC) were stained with propidium iodide staining solution using apoptosis detection kit (Annexin V staining kit). Percentage of MN, MoDCs and PDDCs that present condensed or fragmented nuclei (PI) or the outer membrane (annexin+), (**C**) PDDCs generated from *E. Coli* LPS treatment or infection with Mfa-1^+^ strains (WT, DPG) displays a significantly lower level of surface Annexin V staining compared to starved monocytes and MoDC controls. This significant decrease is lost when cells are pretreated with HIV-1gp120 to block DC-SIGN prior to infection or PDDCs are generated with Mfa-1 deficient strains (MFI, MFB). These results are the mean of three independent experiments (n = 3).

Since the gene expression profile indicated that PDDCs had suppressed levels of apoptosis mediators, we hypothesized that non-canonical differentiation conferred inhibited apoptosis on PDDCs. We tested the hypothesis that pathogen-driven differentiation of non-canonical DC confers extended survival upon these DCs. The immunofluorescence assays of nuclear fractioning and condensing on monocytes (MN), MoDC, and PDDC cultured under serum free conditions *in vitro* exhibited greater susceptibility of MoDCs to become more apoptotic than monocytes or PDDCs (Fig. 1A). We maintained MN, MoDC, and PDDCs in 10% FBS in RPMI for 10 hrs and then the percentage of apoptotic (condensed nucleus) cells was determined. The percentage of apoptotic cells was seen higher in MoDC than PDDC and MN (Fig. 1B). After 24 hours of culture, PDDCs generated by DC-SIGN targeting strains of *P. gingivalis* (WT and DPG-3) showed significantly (***P < 0.001) lower levels of Annexin V than MN or MoDC controls (Fig. 1C). This reduction of Annexin V was ablated when cells were pre-treated with the DC-SIGN-blocking ligand HIV gp120 prior to infection (Fig. 1C).

**Figure 2:**
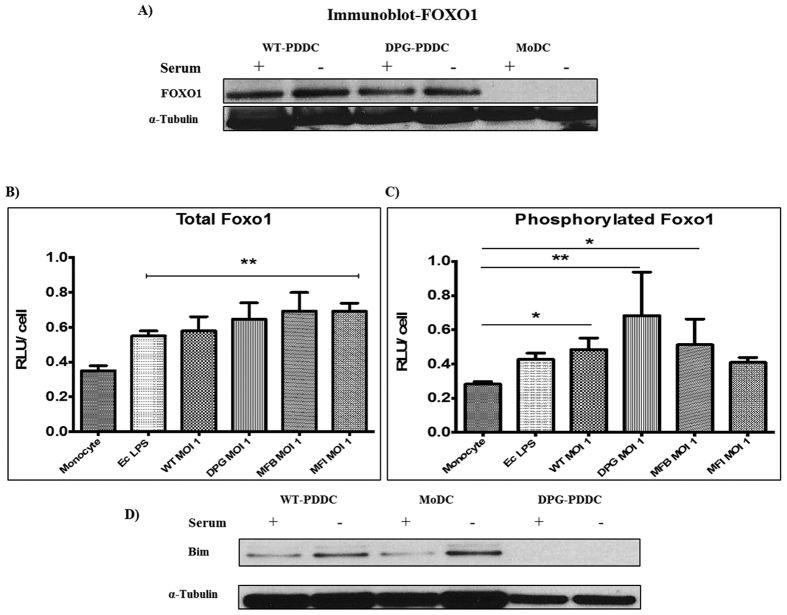
Phosphorylated Foxo1 decreases with PDDC apoptosis rate. (**A**) FOXO1 expression was observed on protein level in PDDCs (WT & DPG) by carrying out Western blot using Anti-FOXO1 Mab. α-Tubulin, loading control (n = 2). The amount of total PDDC Foxo1 (**B**) and phosphorylated Foxo1 (**C**) were measured by whole cell ELISA and normalized to total cell numbers (n = 3) (B) PDDCs generated with *E. coli* LPS or either of the *P. gingivalis* strains have significantly higher levels of Foxo1. (**C**) PDDCs generated by the minor-fimbriae expressing WT and DPG-3 or the non-fimbriated MFB have significantly higher levels of phosphorylated Foxo1 (**D**) DCs were washed in RPMI and then incubated for 10 hrs in 10% FBS in RPMI. DCs were then lysed and Bim was detected by immunoblot. α-tubulin, loading control (n = 2).

FOXO members can transcribe genes encoding pro-apoptotic molecules, including the pro-apoptotic Bcl-2 member Bim, a key regulator of apoptosis. Therefore, under serum free conditions functional Foxo1 plays a pro-apoptotic role in MoDC. Bim, a pro-apoptotic gene controlled by Foxo1, promotes apoptosis of DCs. The immunocytochemistry analysis of WT-PDDCs and DPG-PDDCs (Supplementary Fig. S3), and immunoblot analysis of WT-PDDCs, DPG-PDDCs and MoDCs (control) (Fig. 2A) revealed expression of FOXO1 in PDDCs (WT/DPG-PDDC) but not in MoDCs. Next, PDDCs were generated directly in 96-well plates to perform a whole cell ELISA for measuring total and phosphorylated forms of transcription factor, Foxo1. Akt1 phosphorylates and inactivates the FOXO family of transcription factors including FOXO 1, 3 & 4. Upon phosphorylation by Akt1, FOXO proteins shuttle from nucleus to the cytoplasm where they are no longer transcriptionally active and are retained or marked for degradation. Each of the PDDC groups increased Foxo1 expression per cell compared to untreated monocytes (Fig. 2B). The level of phosphorylated Foxo1 was significantly increased in wild-type (WT) and non-fimbriated (MFB) PDDC groups (**P < 0.01), and even more significantly increased in DC-SIGN targeting (DPG-3) PDDCs (Fig. 2C). These results suggest the crucial role played by FOXO1 in regulating apoptosis in pathogen-DC complex (PDDC).

Having observed the marked increases in levels of functional Foxo1 and phosphorylated Foxo1, we analyzed PDDC expression of Bim by western blot (Fig. 2D). MoDCs normally do not express high levels of Bim, but show a significant increase in Bim expression under serum-starvation signals (Fig. 2D). On the contrary, DPG-PDDCs with or without serum did not express Bim when compared with MoDC controls.

### CD40 contributes to pathogen-derivedDC (PDDC) anti-apoptotic signaling

**Figure 3:**
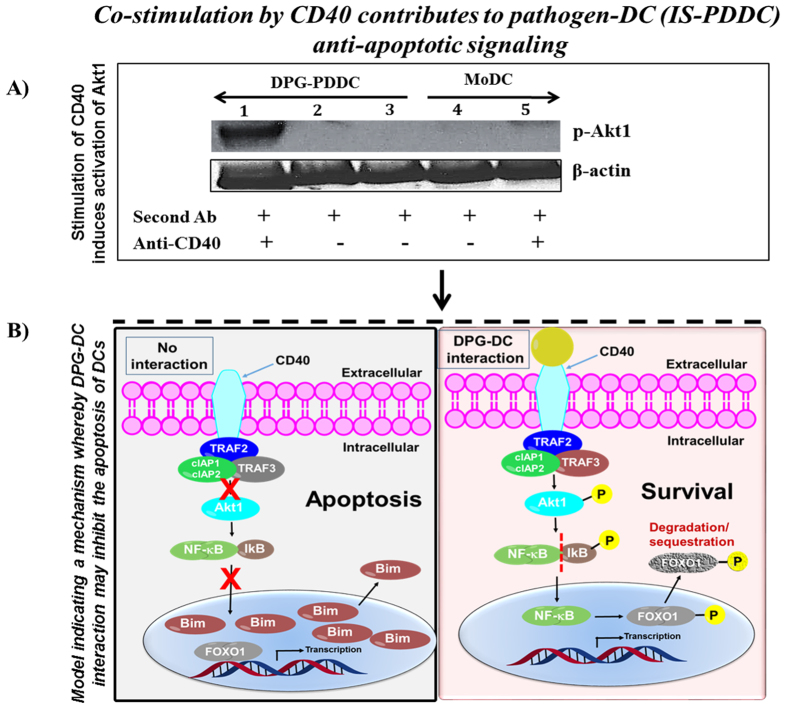
Stimulation of CD40 induces activation of Akt. (**A**) MoDC and PDDC were suspended in 0.1% BSA in RPMI and then treated with anti-CD40 antibody. DCs were then treated with secondary antibody (sheep anti-mouse (Fab) 2 fragment antibody) for 15 min to induce clustering of CD40. The DCs were lysed and phosphorylated Akt1 was detected by using anti-Akt1 antibody. Well (1–3) loaded with DPG-PDDC ; well (4–5) loaded with MoDC. β-actin, loading control (n = 3) (**B**) model indicating a mechanism whereby the DPG-DC interaction may inhibit the apoptosis of DCs. Lower left panel, without DPG-DC interaction, transcription factor NF-kB, associated with its inhibitor IkB, and remains in the cytoplasm. On the contrary, pro-apoptotic factor FOXO1 from nucleus regulates the expression of pro-apoptotic family member Bim. Lower right panel, with DPG-DC interaction, CD40 located at the DPG-DC association induces activation of kinase Akt1. The activated Akt1 leads to, (1) phosphorylation by IkB, which is subsequently degraded, and allowing the translocation of NF-kB to the nucleus. NF-kB may control the transcription of pro-survival genes, and (2) phosphorylates FOXO1 in the nucleus and translocates it to cytoplasm which in turn inhibits the expression of Bim.

Immunological synapse (IS) formation includes receptors such as CD40 (cross-linking agent)^34^. Therefore, we decided to study the ability of CD40 to induce Akt1 activation. The clustering of CD40 was stimulated with specific antibodies, and activation of Akt1 was analyzed. As reported earlier^19^, we detected the cross-linking of CD40 which induced Akt1 phosphorylation (Fig. 3A, Supplemantary Fig. S1A). Our results suggest the anti-apoptotic signaling induced by CD40 cross-linking in DPG-differentiated DC. As reported earlier^35^,^36^, our results confirm the extended survival of DCs (Fig. 3B, Supplemantary Fig. S1A). Our results present the model indicating a mechanism whereby the DPG-DC interaction might inhibit apoptosis of DCs. Figure 3B (left panel) & Supplemantary Fig. S1A, without DPG-DC interaction, transcription factor NF-kB remains associated with its inhibitor IkB, stays in the cytoplasm. On the contrary, pro-apoptotic factor FOXO1 from nucleus regulates the expression of pro-apoptotic family member Bim. Figure 3B (right panel) & Supplemantary Fig S1A, with DPG-DC interaction, CD40 located at DPG-DC association induces activation of downstream effecter of phosphatidylinositol kinase, Akt1. The activated Akt1 leads to, 1) phosphorylation by IkB, which is subsequently degraded, and allowing the translocation of NF-kB to the nucleus. NF-kB may control the transcription of pro-survival genes, and 2) phosphorylates FOXO1 in the nucleus and translocates it to cytoplasm which inhibits the expression of Bim (Fig. 3B).

### Pathogen-derived DCs fail to elicit CD8 T cell activation

**Figure 4:**
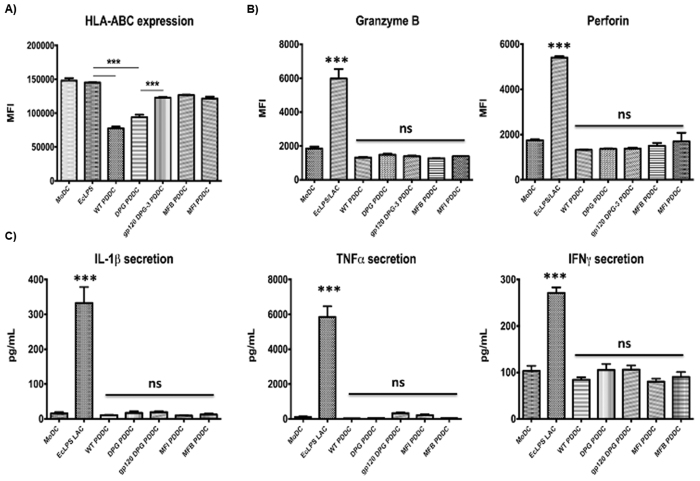
*P. gingivalis* generated PDDCs have inhibited activation of effector CD8 T cell function. PDDCs were generated for 24 hours and then cultured with autologous naïve CD8 T cells (**A**) Surface levels of HLA-ABC on the PDDC groups was analyzed to determine stimulatory capacity. WT and DPG-3 PDDC groups had significantly lower levels of HLA-ABC expression. HLA-ABC was significantly increased with pre-treatment of cells with HIV gp120 to prevent DPG-3 uptake. (**B**) Surface expression of Granzyme B and perforin were analyzed on CD8 T cells after culture. PDDC groups did not drive expression of granzyme B or perforin above baseline levels seen after culture with untreated MoDCs and were much lower than in the presence of *E. coli* LPS and leukocyte activation cocktail (**C**) Analysis of cytokine secretion from supernatants of the co-cultures show that inflammatory cytokines IL-1β, TNFα, and IFNγ are only produced in the presence of *E. coli* LPS and LAC. These results are the mean of three independent experiments (n = 3).

As *P. gingivalis* is an intracellular pathogen and activated CD8 T cells are both important in clearance and observed to increase in periodontal lesions, we investigated the ability of PDDCs to stimulate the activation of CTL effector molecules (Fig. 4). We observed that HLA-ABC expression was relatively high in each respective PDDC group but was significantly lower in PDDC groups differentiated by DC-SIGN (WT, DPG) targeting strains (***P < 0.001) (Fig. 4A). We then cultured each PDDC group with autologous naïve CD8^+^ T cells for 5 days to determine if PDDCs were able to activate a functional cytotoxic phenotype. PDDCs were unable to drive up-regulation of granzyme B or perforin on CD8^+^ T cell surfaces, and only positive control leukocyte activating cocktail resulted in significant increases in both granzyme B and perforin (***P < 0.001) (Fig. 4B). Next, we analyzed cytokine production from the mixed culture for secretion of typical CTL-associated cytokines. IL-1β, TNFα and IFNγ were not significantly up-regulated in any of the PDDC cultures and only expressed with the positive control LAC treatment (Fig. 4C).

### Pathogen-derived DC drive different helper responses in naive CD4 T cells

**Figure 5:**
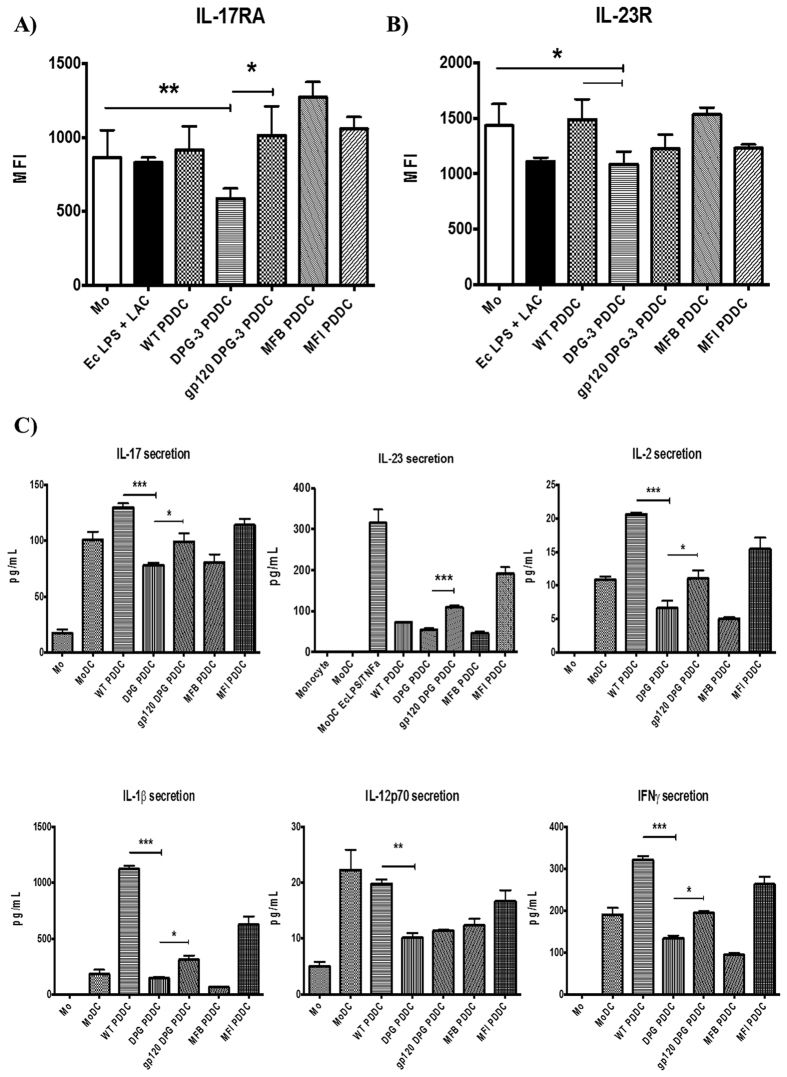
Dampened Th1/Th17 response of PDDCs in absence of *P. gingivalis* major fimbriae. PDDCs were analyzed for surface expression (**A,B**) and cytokine secretion (**C**) of Th1 or Th17 biased inflammatory mediators. (**A**) PDDCs express relatively low levels of surface IL-17 receptor, with minor-fimbriated DPG-3 PDDCs showing a significant reduction in IL-17 receptor expression. The expression is significantly restored when cells are pre-treated with HIV gp120 to prevent DPG-3 uptake. (**B**) PDDCs express relatively low levels of surface IL-23 receptor and DPG-3 generated PDDCs have significantly lower IL-23 receptor than either untreated monocytes or WT generated PDDCs. (**C**) DPG-3 generated PDDCs show a significant decrease of the IL-17, IL-2, IL-1β, IL-12p70, and IFNγ secretion compared to WT generated PDDCs. Secretion of IL-17, IL-23, IL-2, IL-1β, and IFNγ were all significantly increased when cells were pre-treated with gp120. These results are the mean of three independent experiments (n = 3).

**Figure 6:**
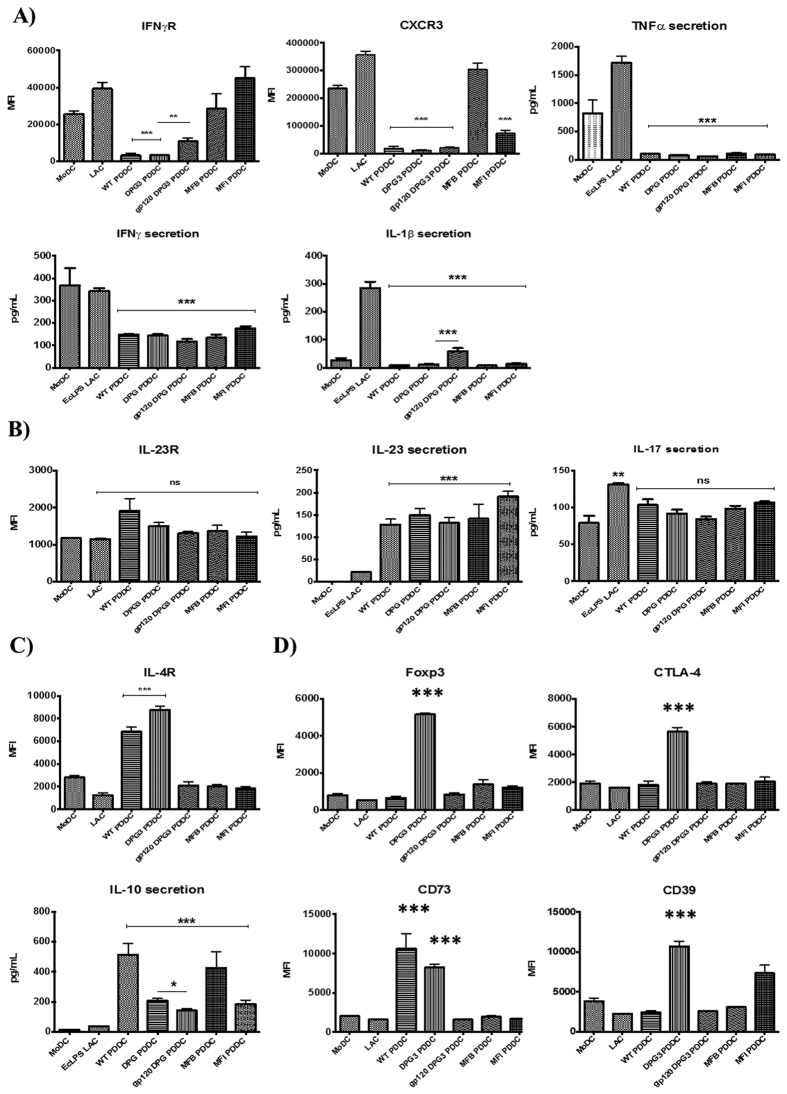
PDDCs are unable to generate robust inflammatory responses from naïve CD4 T cells. PDDC groups were generated for 24 hours and then cultured with autologous CD4 T cells for 5 days. CD4 T cells were analyzed for expression of selected Th1 inflammatory molecules (**A**), Th17 inflammatory molecules (**B**), Th2 molecules (**C**), or Treg molecules (**D**). (**A**) PDDCs generated with *P. gingivalis* minor-fimbriated strains WT and DPG-3 led to a significant reduction of the Th1-biasing IFNγ receptor and CXCR3 on CD4 T cell surface. Expression of IFNγ receptor was significantly increased when initial DPG-3 uptake was blocked with HIV gp120. TNFα, IFNγ and IL-1β secretion were significantly lower in all PDDC group co-cultures compared to the positive control of *E. coli* LPS and leukocyte activation cocktail^70^. IL-1β was significantly increased with gp120 treatment. (**B**) The Th17-biasing IL-23 receptor was significantly increased on CD4 T cells by WT PDDC, but not seen in any of the other PDDC co-cultures. IL-17 secretion was not significantly changed from untreated MoDC co-cultures by any PDDC group co-cultures. IL-23 secretion was significantly increased in all PDDC co-cultures. (**C**) The expression of Th2-biasing IL-4 receptor was significantly increased in WT and DPG-3 co-cultures and significantly decreased when DPG-3 uptake of PDDCs was blocked by gp120. IL-10 secretion was significantly increased in all PDDC co-cultures compared to LAC-treated controls and was significantly decreased in gp120 treatments prior to DPG-3 infection compared to DPG-3 alone. (**D**) CD4 T cells cultured with DPG-3 PDDCs showed a significant increase in Foxp3, CTLA-4, CD39, and CD73 expression. Pre-treatment with HIV gp120 to block DPG-3 uptake significantly reduced expression of each marker. These results are the mean of three independent experiments (n = 3).

Our group has previously shown that *P. gingivalis* minor (Mfa-1) fimbriae are able to generate Th2 helper responses while the major FimA fimbriae generate Th1 helper responses in MoDCs. Thus, we wanted to determine whether these fimbriae interactions that generate PDDCs were also able to drive different Th subsets. We found that PDDCs generated through DC-SIGN ligation (WT and DPG-3) had significant reductions in surface IL-17R and IL-23R (*P < 0.05, **P < 0.01) (Fig. 5A,B) and secreted lower levels of the inflammatory cytokines IL-1β, IL-2, IFNγ, IL-17 and IL-23 (Fig. 5C). In addition, these select PDDCs produced sizeable amounts of anti-inflammatory IL-12p70 (Fig. 5C). This indicated that WT and DPG3-generated PDDCs could function as regulatory DCs. Thus, we cultured each PDDC group with autologous naïve CD4 T cells to determine potential T helper cell responses. To broadly look at T helper subsets, we investigated the expression of typical Th1 receptors IFNγR and CXCR3, and secretion of IL-1β, TNFα and IFNγ^37^,^38^, Th17 receptor IL-23R, and secretion of IL-17 and IL-23, and the Th2 receptor IL-4R^39^,^40^ on naïve CD4^+^ cells after co-culture with PDDC groups for 5 days (Fig. 6). PDDCs generated by DC-SIGN-targeting strains (WT, DPG-3) drive significantly lower levels of IFNγR and CXCR3 expression on CD4^+^ T cells, as well as significant decreases in TNFα, IFNγ, and IL-1β secretion (***P < 0.001, **P < 0.01) (Fig. 6A). Treatment with HIV gp120 before DPG-3 infection and co-culture led to a significant increase in IFNγR expression, but not CXCR3 expression (Fig. 6A). Also, we did not observe significant differences in the ability of PDDC groups to drive IL-23 receptor expression, while all PDDC groups and controls led to significantly more IL-23 secretion than MoDC controls (Fig. 6B). However, there were no significant changes observed in IL-17 secretion from PDDC co-cultures compared to MoDC controls (Fig. 6B). The PDDC groups generated through DC-SIGN, WT and DPG-3, drove significant increases in IL-4 receptor expression and IL-10 secretion from CD4^+^ T cells (Fig. 6C). Strikingly, we found significant increases in Foxp3, CTLA-4^41^, CD73, and CD39^42^ expression on CD4^+^ T cells after co-culture with DPG-3-generated PDDC, indicative of a regulatory-driven response (Fig. 6D). Interestingly, the up-regulation of each analyzed Treg surface marker was inhibited by HIV-1 gp120 blocking of DC-SIGN prior to D’PG-3 infection.

### DPG3-generated DCs drive the generation of inducible regulatory T cells (Tregs)

**Figure 7:**
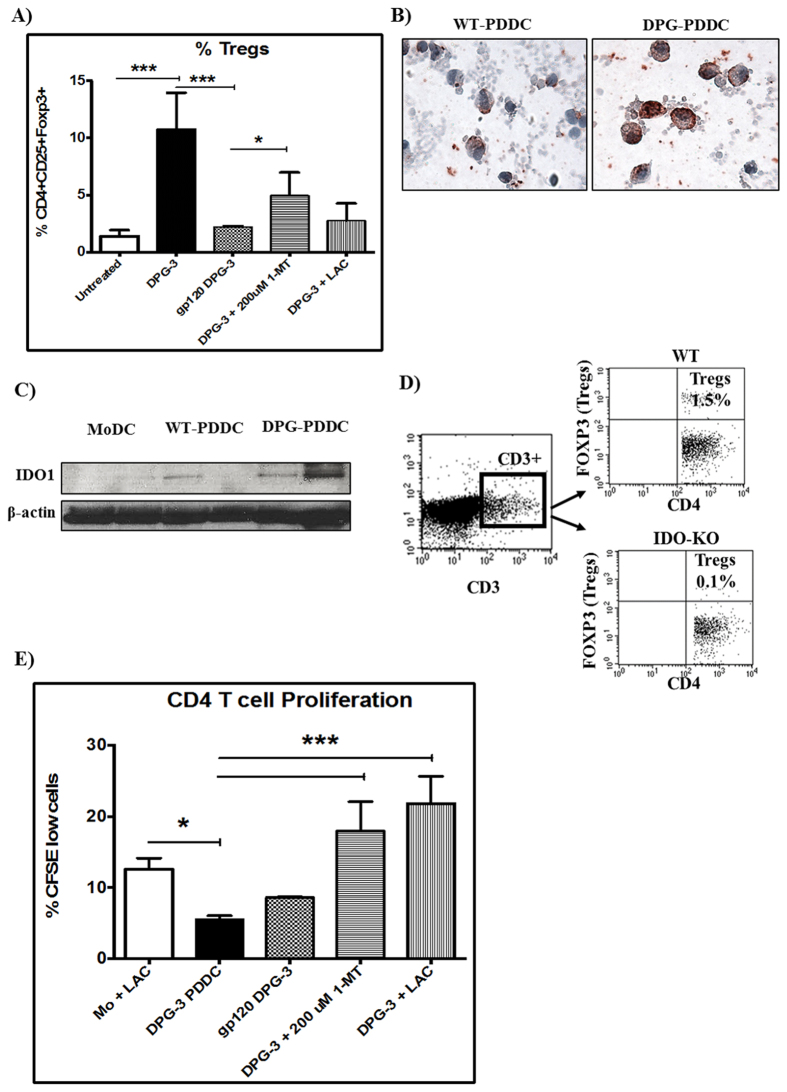
DPG-3 PDDCs induce IDO-dependent regulatory T cell responses from naïve CD4 T cells. PDDC groups were generated for 24 hours and then cultured with autologous CD4 T cells for 5 days. (**A**) Tregs were significantly induced in DPG-3 PDDC co-cultures and this induction was lost with gp120 pre-treatment or in the presence of 1-MT or LAC (n = 3) (**B**) Immunocytochemistry carried out by IDO specific antibody on cytospins of WT-PDDCs and DPG-PDDCs showed greater expression of IDO in DPG differentiated DCs (n = 3). The ICC of IDO expression is well supported and confirmed by, (**C**) immunoblot detection of IDO in WT and DPG-PDDCs. MoDCs and PDDCs were cultured in 10% HI-FBS, cells were lysed by cell lysis solution to extract protein. β-actin, loading control (n = 2). (**D**) IDO plays a crucial role in modulating systemic inflammatory responses by affecting Tregs induction in IDO-KO mouse model of gingivitis. WT (IDO sufficient) mice were able to induce markedly higher level of Tregs (1.5% of CD3^+^ CD4^+^ cells) compared to their IDO-KO counterparts (0.1% of CD3^+^ CD4^+^ cells) after *P. gingivalis* LPS injection (n = 3). (**E**) CD4 T cells were pre-labeled with CFSE to measure proliferation in a parallel experiment. DPG-3 PDDC co-cultures showed a significant reduction in T cell proliferation compared to stimulated MoDC controls. T cell proliferation was significantly increased in DPG-3 PDDC co-cultures in the presence of IDO or LAC (n = 3).

Considering DPG3-generated PDDC were the strongest inducers of Treg markers, we specifically analyzed their ability to generate inducible Tregs from naïve CD4^+^ T cells (Fig. 7). The relative proportions of CD25^+^ Foxp3^+^ CD127^−^ Tregs were assessed in each culture (Fig. 7A). DPG-3 PDDC led to a significant induction of Tregs, which was ablated with gp120, 1- methyl tryptophan (MT), or leukocyte activation control (LAC) treatments (Fig. 7A). Consistent with heightened expression of CD25^+^ Foxp3^+^ CD127^−^ seen in CD4 & DPG-DC co-cultures, we detected greater number of DPG-DCs and CD4^+^ T cell coculture expressing Foxp3^+^ cells on immunocytochemical analysis when compared with MoDC and WT-PDDCs (Supplementary Fig. S4). Furthermore, DPG-3 PDDC led to a significant increase of Foxp3 on CD4^+^ T cells, which was significantly reduced when HIV gp120 treatments blocked DC-SIGN before infection or when cultures were stimulated with LAC (Supplementary Fig. S4). To determine the possible role of immunoregulatory enzyme indoleamine 2, 3-dioxygenase (IDO) produced by DPG3-derived PDDCs in Treg expansion, we utilized the IDO inhibitor (1-MT) in the co-culture. The presence of 1-MT significantly (***p < 0.001) reduced Foxp3 up-regulation on CD4 T cells cultured with DPG3-PDDCs (Supplementary Fig. S4). Due to the inhibition observed with 1-MT treatments, we determined the protein expression of IDO in conventional MoDCs, WT-PDDCs and DPG3-PDDCs. The immunocytochemistry analysis revealed IDO expression in WT-PDDC and more robust expression in DPG3-PDDC (Fig. 7B). We could detect the expression of IDO in DPG-PDDCs PVDF blots when greater amount of protein was loaded on SDS-PAGE both in WT and DPG-PDDCs, but not in conventional MoDCs (Fig. 7C). Further confirming the role of IDO in generation of Treg, IDO-KO mice displayed significant loss of Treg after being challenged with *P. gingivalis* LPS-induced inflammation, compared to WT mice (Fig. 7D). In addition, the role of IDO in the balance of immune response in a periodontal model was confirmed in the IDO-KO mouse model (Supplementary Fig. S5). The loss of IDO increases inflammatory damage and IL-17 production (Supplementary Fig. S5A,B,E) while dampening IL-10 production (Supplementary Fig. S5C,D,F). Next, we determined the overall ability of CD4+ T cells to proliferate in co-culture using CFSE dye dilution. CD4+ T cells had significant decreases in proliferative capacity in the presence of DPG-3 PDDC compared to controls, but proliferation rates were significantly up-regulated in the presence of 1-MT or LAC (Fig. 7E).

### Pathogen-differentiated DC (DC-SIGN expressing ***P. gingivalis*** isogenic mutants) lasting longer in humanized mice

#### Tracking of pathogen loaded MoDC in humanized mice

As DC-SIGN expressing strains (WT & Mfa-1) of *P. gingivalis* were seen less susceptible to become apoptotic as compared to conventionally generated monocytes derived DCs, we validated less apoptotic and long-lasting survival of PDDCs in DC reconstituted humanized mouse. The *P. gingivalis* loaded and CMFDA labeled dendritic cells were traced in huMoDC-NSG humanized mouse.

#### Persistence of signals emitted by CMFDA labeled human monocytes *in vitro*

This study was designed to assess durability of signals emitted from CMFDA labeled CD14^+^ monocytes enriched by RossettSep monocytes enrichment cocktail (StemCell technology, Canada) (Supplementary Fig. S6). There are reports showing use of CMFDA dye for staining various cell types including monocytes, keratinocytes, lymphocytes and stem cells-derived from bone marrow^43^,^44^, to assess the sustenance of signals^45^. We have assessed persistence of signals *in vitro* prior to tracing labeled MoDC in humice. The human monocytes were stained with 10 μM CMFDA following the manufacturer’s recommendations (Supplementary Fig. S6A) and cells (200 K) were cytospin onto slides at different time points (0 to 96 hr). The prepared cells were observed under fluorescence microscope and photomicrographs were taken. We observed the signals lasting more than 96 hrs (Supplementary Fig. S6A), and therefore decided to use CMFDA dye for tracking the labeled monocytes and *P. gingivalis* loaded DCs.

#### Myeloablative treatment required to control the residual innate immune responses of the host

The advent of several new mouse strains with genetic immune deficiencies has largely benefited the development of an experimental laboratory model to study systemic inflammatory disease such as malaria^30^,^31^,^33^,^46^. The immunocompromized mouse NOD/SCID/IL2r^null^ (NSG) mice have depleted adaptive and innate immune responses^47^,^48^,^49^. NSG mice with immune-deficiencies support human cell engraftment, but residual innate immune cells such as monocyte and macrophage play crucial role in the clearance of human cells or pathogens^29^,^50^. As reported^30^ earlier, we validate the importance of phagocytic cells, polymorphonuclears and macrophages, in ingesting and clearing infected and uninfected human cells^30^. While huRBC were seen to be phagocytized by the active macrophages seen on Geimsa stained smears from huRBC reconstituted NSG mice (Supplementary Figure S6C), PMNs and monocyts/macrophages were seen inactive with their pigment (Supplementary Figure S6B). Our results, as others^48^, surface the need to control the residual innate response to achieving sizeable human cell grafting in NSG mice. Since macrophage mediated phagocytosis is responsible for eliminating human RBCs from peritoneum and periphery of xenotransplanted mouse^51^, we used un-sized clodronate-loaded liposome suspension to help facilitate survival of injected human cells (huRBC and huMoDC) by controlling excessively recruited monocytes/macrophages^28^.

#### *Ex-vivo* generated MoDCs pulsed with *P. gingivalis* isogenic mutant(s)

Myeloid DCs differentiated from CD14^+^ monocytes and plasmacytoid DCs derived from plasmacytoid cells present in peripheral lymphoid organs are two known subsets of antigen presenting cells in humans. The huMoDCs are matured by providing exogenous and endogenous stimuli^6^. We report generation of another set of non-canonical and pathophysiological cells from CD14^+^ monocytes which are less susceptible to become apoptotic when compared with conventional MoDCs. These PDDCs were differentiated by DC-SIGN expressing (WT, Mfa-1) *P. gingivalis* strains at 1 multiplicity of infection (MOI) when incubated with positively selected CD14+ 6. The *ex-vivo* generated PDDCs requiring no IL-4 and GM-CSF were characterized and analyzed by flow cytometry^6^.

#### Tracking and traversal of human-monocytes and *P. gingivalis* (WT & DPG) loaded MoDCs in humanized mouse

The NSG mice reconstituted with huMoDC (humanization) received 3–4 injections of clodronate-loaded liposomes to controlling residual innate immune cells in order to achieving sizeable engraftment^52^ before receiving 4.5 million CMFDA (15 μM) labeled monocytes intravenously. We sampled mice at 0 hr, 1 hr, 2 hr, 24 hr and 48 hr after administration of CMFDA labeled human monocytes, stained with CD14, CD83, CD1c & DC-SIGN^+^ antibodies acquired & analyzed to detect presence of monocytes in mouse’s periphery. The absence of monocytes in mouse circulation 2 hrs post-administration drove us to carry out whole body imaging on live animals to trace the labeled monocytes in a reconstituted humanized mouse. Interestingly, we observed the green signals emanating from labeled MN administered to five NSG mice (Supplementary Figure S7) until day 10 post-injection. The animals were positioned well onto instrument platform, and signals were recorded from CMFDA labeled MN at different locations of deep-seated tissues (Supplementary Fig. S7). Next, we decided to trace the DC-SIGN expressing *P. gingivalis* (WT and DPG) loaded MoDCs. The CMFDA (15 μM) labeled MoDCs were pulsed with WT and DPG bacteria to achieve significant loading. 3.8 million MoDCs loaded with WT & DPG-*P. gingivalis* were injected through intravenous route into two humice each. We did not see circulating MoDCs 2 hrs post-administration (Supplementary Fig. S8). Interestingly, we recorded signals through whole body imaging performed on 4 humanized mice (two mice each received WT-P.g. & DPG-P.g. loaded MoDCs) (Supplementary Fig. S8). The green signals were seen lasted for 10 days after injection. The treated animals were positioned well on the instrument’s platform, and signals were recorded by CMFDA-MoDC at different locations in deep-seated tissues (Supplementary Fig. S8). The signals seen in various organs for 10 days post-administration suggest the presence of bacteria in deep-seated tissues.

#### Localization of pathogen (WT & DPG *P. gingivalis*) loaded-MoDCs in humanized mouse

Whole body imaging (WBI) on live animals show the presence of labeled and pathogen loaded DCs in deep-seated organs. However, WBI have limitation in localizing MoDCs. As we wanted to understand evasion mechanism employed by the pathogen to escape host’s immunity, the humanized mice —administered with huMN-CMFDA, WT/huMoDC-CMFDA and Mfa-1/huMoDC-CMFDA were euthanized 4 post-injection, and organs (spleen, liver, heart, kidney, & lungs) were harvested to extract cells onto glass slides to carry out immunofluorescence assay (IFA) (data not shown). The fixed and mounted cells were observed under fluorescence microscope (data not shown). The intense signals were seen from liver, spleen and heart with both WT and DPG loaded MoDCs. The signals recorded on the cells extracted from deep-seated tissues suggest that *P. gingivalis* may have employed an escape mechanism to evade host’s immunity, and thereby sequestered in deep-seated organs, primarily in lungs and heart.

#### Histopathological analysis

Next, we decided to localize *P. gingivalis* pulsed MoDCs in deep-seated tissues (Supplementary Fig. S9) through histopathology. Animals were euthanized on day 4 post-injection and organs (spleen, liver, heart & lungs) were harvested and cryopreserved. The longitudinal sectioning was done by cryotome on different organs collected from humanized mice administered with wild type *P. gingivalis* labeled with CMFDA and loaded onto MoDCs. The sections were fixed and visualized under fluorescent microscope in bright light field. The signals recorded with lungs, heart and spleen are confirmative of the presence of bacteria for longer periods in deep-seated tissues, which validate our hypothesis of escape mechanism employed by pathogen to evade host’s immunity (Supplementary Fig. S9).

## Discussion

Dendritic cells are the most potent antigen-presenting cells with heterogeneous population of myeloid cells that express a wide variety of pattern recognition and lectin receptors. These cells are responsible for cross-talk between innate and adaptive immune responses^44^,^53^. Since their discovery the known role of DCs in both basic and medical sciences has increased to hypersensitivity control^1^ viral infections^2^, transplantation^3^, cancer^4^ and immune regulation^54^. Peripheral blood monocytes acts against the danger signals and rapidly differentiate into DCs^55^ with diverse subsets and functional capabilities. The protective immunity rendered by DCs is based on their interaction with foreign stimulus and their stage of differentiation. The veracity of pathogen-differentiated DC (PDDC) in its contribution towards the regulation of adaptive and non-adaptive immune response is a major question to be addressed. Here, we determined the longevity of PDDCs and their potential role in immune dysregulation in context of interaction with a chronic pathogen. Present study sequentially analyzed the ability of PDDCs to trigger a broad range of adaptive and innate immune responses.

Our observation that DC-SIGN ligation leads to reduced apoptosis in MoDCs^6^ prompted us to investigate anti-apoptotic signaling involved in PDDCs differentiated through DC-SIGN ligation. First, we utilized a gene-expression microarray to elucidate expression differences of various biomarkers including pro-and anti-inflammatory cytokines, chemokines, accessory molecules, pro-and anti-apoptotic signaling factors/genes, co-stimulatory molecules, and regulatory T cells associated markers in PDDC and MoDCs. We saw a marked inhibition in the expression of apoptotic markers with PDDCs compared to MoDCs. Specifically, alterations in Foxo1, Bim, BIRC5 (survivin), CD40 and STAT1 stood out as they are important, either directly or indirectly, in regulating DC lifespan^19^. Interestingly, phosphorylated Foxo1 and CD40 signaling molecules confer extended survival on DCs by regulating the expression of pro-apoptotic gene, Bim^56^. The expression of Bim was depleted in PDDC differentiated by the DC-SIGN-targeting (WT and DPG-3) strains. The inhibited susceptibility of PDDCs towards apoptosis than MoDCs drove us for transcript level experimental confirmation. All PDDC groups had elevated total and phosphorylated Foxo1 compared to monocytes, while PDDCs differentiated through DC-SIGN ligation (WT and DPG-3) showed greater levels of inactive (phosphorylated) Foxo1. Phosphorylation not only leads to Foxo1 degradation, but also prevents expression of pro-apoptotic Bcl protein, Bim (Bcl2-l1)^57^. We noticed inhibited expression of pro-apoptotic gene, Bim in PDDC groups, and subsequent reduction of apoptosis via surface Annexin V staining and immunofluorescence assay. The percentage of MoDC undergoing apoptosis was seen significantly reduced in PDDCs generated by DC-SIGN expressing and targeting isogenic mutant of *P. gingivalis* under serum free conditions.

The altered rates of apoptosis seen in DC-SIGN-generated PDDCs led us to further investigate the potential of PDDCs in generating immune responses. The priming of CD8^+^ T cells by DCs is a critical step to establishing specific cytotoxic lymphocyte (CTL) function^17^. As chronic infection by intracellular pathogens often subverts or inhibits priming, we decided to determine the CD8^+^ T cell activation directed by PDDCs. We observed that HLA-ABC expression was significantly lower in PDDCs than experimental controls. Also, PDDC were unable to drive the expression of perforin or granzyme B on naïve CD8^+^ T cells. The lack of priming was associated with moutning of lower levels of inflammatory cytokines necessary for CTL activation. Further investigation of PDDC differentiation and maturation status could provide insight for therapeutic targets to elicit PDDC maturation and effective activation of functional CTL.

As others^18^, we explored the microbial stimulation driven DCs differentiation and their priming of Th cells *in vivo* by culturing PDDCs with autologous naïve CD4^+^ T cells to determine both the expression of surface molecules, and pro-and anti-inflammatory cytokine secretion from the broad range of Th subsets. Interestingly, we found that T cell differentiation was dictated by initial pathogen interaction to generate PDDC. The presence of *P. gingivalis* major fimbriae (MFI strain), previously shown to elicit Th1 cytokine bias^23^, led to the production of Th1 cytokines and weak Th1 cell activation. Conversely, the presence of *P. gingivalis* Mfa-1 (WT and DPG-3) promoted Th2 response, but with weak Th1 response. Interestingly, the DPG-3-generated PDDC, generated via DC-SIGN interaction, led to a strong induction of Treg-associated markers shown by the increased expression of Foxp3, CTLA-4, CD39, and CD73. The immature or regulatory DCs reportedly produce indoleamine 2,3-dioxygenase^58^ and therefore we hypothesized that regulatory T cells produced by PDDCs (WT, DPG-3) could be IDO dependent. The higher rates of necrotic and apoptotic cell death after *P. gingivalis* LPS challenge of IDO-KO mice in comparison to WT mice confirm our investigation of extended survival of PDDC. Tregs produced by DC-SIGN-generated PDDCs were blocked by the ablation of IDO using an IDO inhibitor (1-methyl tryptophan) validating the production of IDO by PDDCs. In addition, flow cytometry analysis of IDO-KO mice showed a marked decrease in Foxp3^+^ Tregs when challenged with LPS *P. gingivalis* than WT mice. Our results suggest crucial role played by IDO in modulating inflammatory responses via regulatory T cell activation. IDO is produced by DC at various stages of development and affects chronic disease. Further, IDO-dependent generation of Tregs might be a potential mechanism of PDDC-mediated immune regulation. Natural Tregs are thought to play pivotal role in the pathogenesis of chronic periodontitis and help *P. gingivalis* evade host’s defense by their immunosuppressive and immune modulation abilities^59^. The regulatory cytokines are also seen up-regulated in deep-seated tissues during CP^60^,^61^. Although both natural and inducible Tregs have been shown to play crucial role in the pathogenesis of chronic infections^62^, factors involved in recruitment and in the maintenance of Treg responses are unknown. Our data, as others^35^, suggests a mechanism in which dysregulated DCs are promoting a robust inducible Treg response. Foxo1 deficiencies have been reported to result in defective Treg responses which are unable to rescue mice from autoimmune disease^63^, and aid in the activation of Foxp3 in inducible Tregs^64^. The FOXO transcription factors were discovered to play an essential role in specifying the program of T cell differentiation in the pathway leading to development and function of Tregs. Further, development of both natural & Tregs required FOXO transcription factors^59^,^60^.

Present study illustrates the differentiation ability of adaptable myeloid monocytes into dysregulated DCs and direct T-cell differentiation. We have previously shown that PDDC are immature and retain their phagocytic capacity, however, immature state of PDDCs might lack antigen processing and presentation on MHC molecules. Th cells play an instrumental role in controlling infections, and inability of PDDC to drive robust responses may help long-lasting survival of intracellular pathogen, establishment of chronic infection, and systemic dissemination of intracellular pathogen. The current study shows nature of pathogen-DC interaction, to generate PDDC by DC-SIGN ligation might subsequently drive polarization of naïve T cells into Tregs. We strongly believe that ability of PDDC to arise quickly after infection and survive during disease progression may have a critical role in generation and maintenance of immune regulation and dysfunction.

As PDDCs were shown to become less apoptotic, and confer extended survival, we tried to decide the fate of pathogen in huMoDC reconstituted immunodeficient mouse (huMoDC-NSG). As monocyte and/or MoDCs loaded with *P. gingivalis* were not seen in the circulation of reconstituted humanized mouse 48 hr post-injection, we traced the path and residence time of CMFDA labelled DCs in deep-seated organs through whole body imaging. Interestingly, signals emitted from CMFDA labeled MN/MoDCs were seen until day 10 post-injection, which supported our hypothesis of escape mechanism that bacteria may have employed to evade host’s residual innate immunity. Furthermore, we validated our observation by carrying out immunofluorescence assay with different organs collected on day 4 post-injection. The results suggesting the extended residence of the pathogen in different organs primarily liver, heart and spleen were supported by histopathology conducted on cryo-preserved tissues. We believe sequestration of *P. gingivalis* in the heart suggests a correlation between periodontal infections and cardiovascular diseases. The long-lived surviving nature of regulatory DCs with persistent intracellular pathogen was confirmed and validated in NSG humanized mouse. To our knowledge, we are the first group to show the long-lived survival of *P. gingivalis in vitro* as well as in a reconstituted humanized mouse. Here, we demonstrate the ability of PDDC to drive differential adaptive immune responses based on their interaction with pathogen. We observed the ability to elicit Th1, Th2, or Treg responses from PDDC based on their route of pathogen interaction. Further studies are needed to elicit the intrinsic DC factors that lead to promotion of various T cell responses. As DCs have proven to be promising candidates for specific, immunogenic, and long-lived vaccinations, this work introduces the ability to further fine tune DC vaccines to promote specific helper T cell subsets. In terms of regulatory microenvironment created during chronic infections, PDDC-based generation of Tregs could provide a unique therapeutic target to promote pathogen clearance while maintaining natural immune regulation. The PDDCs driven production of Tregs may be used to develop therapeutic interventional approaches for diseases such as cancer. Our observation that DC-SIGN interaction confers longevity to PDDCs supports the existence of this mechanism *in vivo.* Our results show that a novel subset of cell, PDDCs, are long-lived and dys-regulated immune cells with a unique ability to modulate immunity based on very specific host-pathogen interaction.In conclusion, we have identified an important new pathway whereby chronic low-grade (oral) infections can negatively influence immune surveillance through host modulation. This may prove to be an important route explaining how relatively asymptomatic diseases such as periodontitis contributes to cancer risk.

## Material and Methods

### Chemicals and reagents

GM-CSF and IL-4 were obtained from Gemini Bioproducts. Fluorescent dye for cell labeling: CFSE was obtained from eBiosciences, San Diego, USA. The cell tracker CMFDA (C7025) was procured from Life technologies, USA. De-complemented FBS, RPMI and X-Vivo 15 were procured from Lonza, USA. EasySep™ Human CD14 Positive Selection kit, and Human monocyte enrichment cocktail (RosetteSep) were purchased from StemCell Technologies, Canada. RNeasy mini kit obtained from Qiagen. High capacity cDNA reverse transcription kit, custom-designed TaqMan array fast plates and TaqMan fast universal PCR master mix (2x) were obtained from applied biosystems, USA. The Poly-l-Lysine coated dishes were obtained from BD Biocoat. The ELISA kit for in-cell western analysis for phospho FKHR (FOXO1) was obtained from Active Motif. Annexin V-FITC and propidium iodide staining kit was procured from eBioscience. The leukocyte activating cocktail was procured from BD biosciences. The anti-human CD4^+^ and CD8^+^ were procured from eBioscience. CD83, CD14, CD209, and CD1c were purchased from eBioscience & Miltenyi Biotech. Regulatory T Cell staining kit, CD127, CD152, CD73, CD39, CD317, Perforin, Granzyme, HLA-ABC were obtained from eBioscience. ProcartaPlex Human cytokine and chemokine panel 1A was purchased from eBioscience. CD183, CD194 and CD132 were from BD Bioscience. IL-23R was obtained from Antibodies online. The cell lysis buffer and protease inhibitor PMSF was from cell signaling. Anti-FOXO1 Mab (C29H9), Anti-rabbit IgG-HRP, normal goat serum, signal stain (R) Ab diluent, Signal stain (R) boost IHC detection reagent (HRP-Rabbit) and Rabbit (DA1E) isotype control were obtained from Cell signaling. Α-Tubulin (PA5-29135), β-Actin (MA1-91399), anti-Bim polyclonal antibody (PA5-20089) and Ultra-vision plus detection system (DAB plus substrate) were obtained from Thermo Scientific. Anti-IDO1 antibody was obtained from LSBio LifeSpan BioSciences, USA. Mouse anti-Akt1 antibody (558316), HRP goat anti-mouse IgG (554002) and Rabbit polyclonal IgG (27478) were obtained from BD Pharmingen. The anti-CD40 monoclonal antibody was obtained from Thermo Scientific, USA. The goat anti-mouse IgG H& L (HRP) and anti-Foxp3 Polyclonal antibody (ab10563) were procured from Abcam. Sheep anti-mouse IgG (whole molecule, A3563) was obtained from Sigma Aldrich. Pierce ECL Western blotting substrate was obtained from (Pierce/Thermo Scientific). Vecta shield fluorescence mounting medium was from Vector laboratories, CA, USA. LPS was purchased from InvivoGen (San Diego, CA). The clodronate-loaded liposomes suspension was provided by N. Van Rooijen, Netherlands.

## Animal ethics committee approval

All animal procedures were carried out in compliance with American animal welfare laws and regulations. All procedures were reviewed and approved by Georgia Regents University, Institutional Animal Care and Use Committee (IACUC Protocol number: 2013−0586)

### Mice

IDO-deficient (IDO1-KO) and IDO-sufficient (IDO-WT) having B6 (C57/BL6) backgrounds, mice were purchased from Jackson Laboratories, (Bar Harbor, ME). Four to six week old male and female NOD/SCIDIL-2Rγ−/− (NSG/NOG) mice were procured from Taconic, USA. These immunodeficient/transgenic mice were housed in sterile isolators and supplied autoclaved tap water with a γ-irradiated pelleted diet ad libitum. They were manipulated under pathogen free conditions using laminar flux hoods.

### Bacterial culture growth conditions and MoDC infection

Wild-type (WT) Pg381, which expresses both minor (mfa-1) and major (fim A) fimbriae, isogenic major fimbria-deficient mutant DPG3, which expresses only the minor fimbriae (mfa-1^+^/fimA^−^), the isogenic minor fimbria-deficient mutant MFI, which expresses only the major fimbriae (mfa-1^−^/fimA^+^) and the double fimbriae mutant/naked MFB (Pg mfa-1^−^/fimA^−^) were maintained anaerobically (10% H_2_, 10% CO_2_, and 80% N_2_) in a Coy Laboratory vinyl anaerobic system glove box at 37 °C^65^ in Acumedia Wilkins-Chalgren anaerobe broth. Erythromycin (5 ug/ml) and tetracycline (2 ug/ml) were added according to the selection requirements of the strains^66^. For calculation of bacterial Multiplicity of infection (MOI), the bacterial suspension was washed twice in PBS and was re-suspended in PBS to an OD at 660 nm of 0.11, which previously determined to be equal to 5 × 10^7^ CFU^67^. The MoDC were pulsed with *P. gingivalis* strains at 1 MOI for 24 hrs. The low MOI values were used to mimic a natural blood mDC infection observed in CP patients^8^,^68^ as well as to avoid overwhelming the host response.

### Cultivation and phenotypic characterization of pathogen differentiated and monocyte-derived DCs

The Human Assurance Committee (HAC) at Georgia Regents University approved all IRB protocols involving human subjects. All human subject studies conducted conform to the principles of the Declaration of Helsinki. The informed consent was obtained from all healthy volunteers before the study was commenced. The conventional MoDCs were generated *ex vivo* as described elsewhere^55^ with slight modifications. Briefly, monocytes were isolated from total PBMCs using negative selection of CD14^+^ cells (StemCell RosetteSep) as well as using human monocyte enrichment cocktail by positive selection. Monocytes were then evenly split in 6-well plates at a concentration of 1–4 × 10^5^ cells/ml in RPMI 1640 containing 10% de-complemented-FBS and antibiotic/anti-mycotic and immediately infected with all isogenic *P. gingivalis* mutants (DPG-3, MFI, MFB) and wild type at 1 MOI to generate pathogen derived DCs^6^. The *ex-vivo* generation of conventional PDDCs was carried out by cultivating selected CD14^+^ monocytes in presence of GM-CSF and IL-4 (Gemini Bio-products) for 6 days. The initial infection was deemed day zero and differentiation was determined and cells were phenotyped at each time point by collecting and analyzing scatter graph to confirming the immature DC phenotype (CD14^low^ CD83^−^ CD1c^+^ DC-SIGN^+^). The cell surface markers of DCs were evaluated by four-color immunofluorescence staining with the following mAbs: CD83, CD14, CD209, and CD1c.

### Genomic microarray (qRT-PCR) of *P. gingivalis* differentiated DCs

Analysis of 48 genes (Table 1, S1 Figure) expression in PDDCs (WT, DPG, MFI, and MFB-DC), MoDCs and monocytes was performed using reverse transcription PCR (RT-PCR). Total RNA was isolated from cells using RNeasy kit at 0 hr MN, monocytes cultured for 6 hrs, monocytes incubated with isogenic P.g. mutants at 1MOI for 6 hrs, and MoDCs. Briefly, cDNA was synthesized by using 1.1 μg RNA through a reverse-transcription reaction (Applied biosystems). Qualitative RT-PCR was performed on TaqMan array fast plates using TaqMan fast universal PCR master mix (2x). The 2^−ΔΔCt^ method was used to calculate fold regulations. The fold regulation of the gene in each group was calculated relative to its expression in the controlled samples for that gene, and was calculated by using GAPDH as the house-keeping control.

#### FOXO1 activity of PDDC

The levels of total and phosphorylated FOXO1 in PDDCs were measured using a whole cell ELISA following manufacturer’s recommendations for suspension cultures and colorimetric assays (Active Motif 48160). The isolated monocytes were seeded at 20,000 cells/well in a 96-well plate and infected with all strains of *P. gingivalis* for 24 hours and other set of cells matured with a cocktail of *E. coli* LPS and TNFα for 24 hours. Plates were then read on an Epoch (BioTek) plate reader at 450 nm with a correction wavelength of 655 nm. The readings were then normalized to total cell counts in each and every single well by staining with crystal violet and reading at 595 nm.

#### Annexin-V staining of PDDCs

Annexins are a family of calcium-dependent phospholipid-binding proteins, which bind to phosphatidylserine (PS) to identify apoptotic cells. In healthy cells, PS is predominantly located along the cytosolic side of the plasma membrane. Upon initiation of apoptosis, PS loses its asymmetric distribution in the phospholipid bilayer and translocates to the extracellular membrane, which is detectable with fluorescently labeled Annexin V. Annexin V staining, paired with 7-AAD or PI is widely used to identify apoptotic stages by flow cytometry. The pathogen differentiated DCs generated after incubation of MN with *P. gingivalis* after 24 hours were analyzed for Annexin V-FITC and propidium iodide (PI) using a cell death detection kit for flow cytometry following manufacturer’s recommendations.

#### Flow cytometry gating and statistical analysis

Gates were chosen using suspension cell forward scatter and side scatter characteristics. Non-adherent MoDCs typically fall within fsc-h 300–600 and ssc-h 100–200 (x1000). Undifferentiated monocytes, lymphocyte carry-over, and debris were excluded from MoDC analysis. Monocytes displayed 95–99% differentiation into MoDCs by day 5 of culture. CD1c^+^ DC-SIGN^+^ cells were chosen for analysis of PDDCs, and statistical analyses for all experimental samples were compared to each other using ANOVA on the means from at least 3 independent flow experiments using Graph Pad Prism 6 and significance is denoted by asterisks within figures where *p < 0.05, **p < 0.01, and ***p < 0.001.

#### Immunofluorescence assay (IFA) and immunocytochemistry (ICC)

IFA was performed on cells aggregated on a glass slide using cytospin (Thermo Scientific, USA). Briefly, cells were plated at 37 °C for 4 minutes at 1000 rpm onto slide by cytospin. The cells were fixed in 4% paraformaldehyde in PBS (10 min at room temperature). The cells were stained with DAPI to estimate % apoptosis. The PBS washed cells were mounted with fluorescence mounting medium. The cells were observed under fluorescence microscope using DAPI channel.

Immunocytochemistry was carried out on WT and DPG differentiated DCs following manufacturer’s recommendations (Cell signaling, mAb#2880). The cells were cytospin onto the slide for 4 minutes at 1000 rpm. The cells were fixed in 4% paraformaldehyde in PBS (10 min at room temperature) followed by three times washing in dH_2_O for 5 minutes each. The cells were incubated in 3% hydrogen peroxide for 10 minutes and cells were washed twice in dH_2_O for 5 minutes. The cells were then blocked with 100–400 μl blocking solution (TBST/5% normal goat serum) for 1 hr at room temperature. The cells were incubated overnight with 100–400 μl FOXO1 rabbit mAb diluted in Signal Stain antibody diluent at 4 °C. The cells were washed three times with wash buffer (TBST) for 5 minutes and cells were covered with 1–3 drops of boost detection reagent in a humidified chamber for 30 min at room temperature. The cells were washed again and 100–400 μl DAB plus substrate was applied for 5–15 minutes to achieve acceptable staining intensity. After washing with water, cells were counter-stained with hematoxylin following manufacturer’s instructions. The mounted cells were and observed under microscope (Nikon instruments) and photomicrographs were taken. ICC experiments were carried out to see an expression of Fox 1 on cytospin of MoDC, WT and DPG-DCs following manufacturer’s recommendations.

### Immunoblot analysis

To analyze total and phosphorylated proteins, DCs (1.4 × 10^6^ cells) were solubilized in lysis buffer, and 1 mM protease inhibitor (PMSF) was added immediately before use. All plated cells (both adhered and in suspension) were solubilized in ice cold 100 μl cell-lysis buffer mixed with 1 mM PMSF, incubated on ice for 5 min, sonicated briefly following manufacturer’s recommendations. The lysates were clarified by centrifugation at 14000 rpm for 10 min and pellets were discarded. The supernatant was used to carry out immunoblot analysis. After extraction, all samples were separated by SDS-PAGE and immunoblotted. For the immunoblot analysis, samples fractionated by SDS-PAGE were electrotransferred to membranes. After blocking with 5% nonfat milk protein in TBST or PBST (0.1% or 0.2% Tween-20), membranes were incubated with antibodies dissolved in TBS plus 0.1 or 0.2% Tween-20 solution and 5% BSA. Subsequently, the membranes were incubated with suitable peroxidase-conjugated secondary antibodies (Cell signaling and Abcam), and immunoreactive bands were visualized using ECL reagents (Pierce).

### PDDC-T cell co-cultures

CD4^+^ and CD8^+^ T cells were isolated using RoboSep automated negative selection and purity confirmed by staining with anti-human CD4^+^ and anti-human CD8^+^, respectively. The generated PDDC were retrieved either at 24 or 48 h post-infection and after 24 h induced maturation with *E. coli* LPS and TNFα was imposed. The negatively selected naive T cells were counted and added to PDDC groups at a ratio of 1:3 for 5 days. The positive control group was a co-culture that was treated with leukocyte activating cocktail (*E.coli* LPS/TNFα). After culture conditioning, cells were collected and fixed with 4% paraformaldehyde and stained with the following anti-human antibodies for effector subsets, Th1 subsets: CD183 (CXCR3) and CD119 (IFNγR); Th2 subsets: CD194 and CD132; Th17 subsets: CCR6 and IL-23R; and Treg subsets: Regulatory T Cell staining kit, CD127, CD152, CD73, CD39 and CD317. The CTL activation was measured by staining with the following anti-human antibodies, on CD8 T cells: Perforin and granzyme B and on PDDCs: HLA-ABC.

### Preparation of host for human MoDCs grafting: immunomodulatory agents and suppression of innate immunity

Numerous attempts were made to increase the success rate of the grafting of human cells such as infected and uninfected huRBC. The clodronate loaded liposome suspension was injected through intraperitoneal (i.p.) route in order to reduce the number of tissue MP as described elsewhere^28^,^29^. The immunodeficient NSG mice were retro-orbitally injected with 6 million huMoDCs reconstituted in 250 μl RPMI suspension of huMoDC every 3 days to ensure a satisfactory proportion of huMoDC (chimerism) at the time of tracking and traversal of *P. gingivalis.* Simultaneously, NSG mice were administered 0.1 ml of un-sized dichloromethylene diphosphonate (Cl2MDP) encapsulated in liposome (clo-lip) diluted in 0.3 ml RPMI was intraperitoneally injected. Four injections at 2–3 day intervals were given prior to tracking the *P. gingivalis* loaded onto huMoDCs.

### Monocyte and MoDC staining with cell tracker dye CMFDA, and whole body imaging on live animals

Briefly, monocytes were isolated from total PBMCs using negative selection of CD14^+^ cells (Stemcell RosetteSep) and using human monocyte enrichment cocktail by positive selection. The enriched cells were counted by Hemocytometer^69^ and flow cytometry. The persistence of signals emitted from CMFDA labeled monocytes were assessed *in vitro*. The cells (200,000) were harvested by centrifugation, and cell pellet was re-suspended gently in pre-warmed CellTracker™ CMFDA (Life technologies-C7025, USA) 10 μM working solution (according to manufacturer’s recommendations). The stained cells were incubated 15–45 minutes under growth conditions appropriate for the particular cell type. The cells were centrifuged and excess of dye was removed, and culture medium was added. The incubated cells were cytospin onto slides at 0 hr, 24 hr, 48 hr and 96 hr to determine sustenance of dye signals.

Mice were further modulated for their residual innate immunity by treating three to four times with clodronate-loaded liposomes (100 μl reconstituted in 300 μl RPMI) through intraperitoneal route at every 3 days interval prior to injecting CMFDA labeled human monocytes and MoDCs. Each mouse received 4.5 million monocytes labeled with 15 μM CMFDA, and cells were tracked in NSG mice through whole body imaging (Xenogen, *in vivo* spectrum, USA) instrument. The animals were anesthetized and were positioned on the platform of the imaging machine prior to imaging was carried out on the treated animals. The *ex-vivo* generated and CMFDA labeled MoDCs were pulsed with WT and Mfa-1 isogenic mutants of *P. gingivalis.* The stained and P.g. loaded MoDC were intravenously administered into the reconstituted humanized NSG. Each mice was intravenously injected with 3.8 million *P. gingivalis* pulsed and CMFDA labeled MoDCs. The signals emanated from labeled monocytes and MoDCs showed persistence in deep-seated organs instead of mouse circulation, and lasted for more than 10 days post-injection.

### Immunofluorescence assay & histopathology

The microscopic analysis was done on the mice euthanized on day 4 post-administration of CMFDA labeled monocytes, and CMFDA labeled & P.g (WT and Mfa-1) loaded MoDCs. The organs (spleen, liver, heart, kidney & lungs) of the euthanized treated animals, and minced organs slides were prepared, fixed and viewed under microscope. The tissues were collected and cryopreserved in liquid nitrogen, and were then processed for sectioning with Shandon cryotome E cryostat (Germany) to get 5 μm thick sections. The sections were fixed with 4% paraformaldehyde, washed three times with PBS, and photomicrographs were taken under the microscope.

## Additional Information

**How to cite this article:** Tyagi, R. K. *et al*. Human IDO-competent, long-lived immunoregulatory dendritic cells induced by intracellular pathogen, and their fate in humanized mice. *Sci. Rep.* **7**, 41083; doi: 10.1038/srep41083 (2017).

**Publisher's note:** Springer Nature remains neutral with regard to jurisdictional claims in published maps and institutional affiliations.

## Supplementary Material

Supporting Information

## Figures and Tables

**Table 1:** *P. gingivalis* wild type and isogenic fimbriae deficient mutants.

Strain	Description	Predominated receptor targeted on DCs
Pg381	Wild-type (381), which expresses both minor (Mfa1) and major (FimA) fimbriae.	DC-SIGN and TLR2
Mfa1^+^	Pg Isogenic major fimbriae-deficient mutant DPG3, which expresses only the minor fimbriae (Mfa1).	DC-SIGN
FimA^+^	Pg Isogenic minor fimbriae-deficient mutant MFI, which expresses only the major fimbriae (FimA).	TLR2
MFB	Isogenic double fimbriae mutant MFB, which lacks both minor and major fimbriae.	TLRs

**Table 2:** Monocyte-derived DCs (MoDCs) and Pathogen (*P. gingivalis*) differentiated dendritic cells (PDDCs) regulate expression of various markers relative to monocytes (Fold regulation shown in selected genes which are expressed on MoDC and PDDC).

Gene markers	Fold change in gene expression in MoDC	Relative fold change in gene expression in pathogen (*P. gingivalis*) differentiated DC			
		WT-PDDC (Wild type)	DPG-PDDC (Minor Fimbriae)	MFI-PDDC (Major Fimbriae)	MFB-PDDC (Double mutant)
CD80	2	1	11.2	8.9	8.3
CD83	3.4	0.9	1	4.1	2.1
CD86*	8.15	0.3	2.8	0.2	0.2
CD40*	17.85	1.1	4.3	5.85	4.6
CD275	2.7	0.55	4.4	1.05	0.2
CD274	10.5	11	23	123	65
CD39	16.56	0.494	1.2	0.95	0.31
IL-1β*	0	4.65	41.9	16.55	8.7
IL-6*	0	16	48	312	231
IL-10*	2.15	35.6	32.1	65.35	62.05
IL-12β	1.3	34.85	5.5	34.4	93.7
IFNγ	0	1.9	0	14.6	41.71
TGFBR2	8.5	0.55	1.5	0.52	0.245
SOCS 1	10	6	5	37	123
SOCS 3	0.1	2.1	5.2	14.1	511.05
STAT1	4.25	1.1	2.45	1.05	2
IDO1*	0.05	1.335	5.85	7.125	36.85
FOXO1*	3.15	1.05	4.35	1.05	0.55
BCL2	1	1.75	0.6	2.1	1.05
BCL2l1	8.15	2.2	1.15	4	2.15
TNFRSF10B	8.1	1.05	1.05	4.2	2.1
BIRC5*	3.55	2.35	15.2	4	4.2
ADORA2B	3.75	0.5	22.25	1	0.55
TNFα	0.095	4	0.3	16	16

Monocytes were isolated using human monocyte enrichment cocktail (RosetteSep), and were cultured in presence of growth factors (GM-CSF and IL-4) for 6 days to generated MoDCs, and 200 K-400 K monocytes/well distributed in a six-well plate, and infected immediately with wild type, minor (mfa-1) fimbriae, major (MFI) fimbriae and double (MFB) isogenic mutant(s) of *P. gingivalis* at 1 MOI. MoDCs and PDDCs were confirmed for their immature DC phenotype (CD14^low^CD83^−^CD1c^+^DC-SIGN^+^) on day 6 and 6 hrs post-infection respectively. The gene expression in MoDCs and PDDCs relative to monocytes was performed by using a custom-designed PCR array plates procured from Life technologies. All markers were designed in triplicates on array plates. The fold change (in gene expression) value greater than one indicates a positive value or an up-regulation of gene expression, and less than one indicates a negative value, or down-regulation of gene expression. Asterisks denote significance difference in genes in MoDCs and DPG differentiated DCs.

## References

[b1] Blazquez A. B., Cuesta J. & Mayorga C. Role of dendritic cells in drug allergy. Current opinion in allergy and clinical immunology 11, 279–284, doi: 10.1097/ACI.0b013e3283489bab (2011). 21659861

[b2] Dejnirattisai W. . A complex interplay among virus, dendritic cells, T cells, and cytokines in dengue virus infections. Journal of immunology (Baltimore, Md.: 1950) 181, 5865–5874 (2008). 10.4049/jimmunol.181.9.5865 18941175

[b3] van Kooten C. . Dendritic cells as a tool to induce transplantation tolerance: obstacles and opportunities. Transplantation 91, 2–7 (2011). 21452405 10.1097/tp.0b013e31820263b3

[b4] Palucka K., Ueno H., Fay J. & Banchereau J. Dendritic cells and immunity against cancer. J Intern Med 269, 64–73 (2011). 21158979 10.1111/j.1365-2796.2010.02317.x PMC3023888

[b5] Garg N. K., Dwivedi P., Prabha P. & Tyagi R. K. RNA pulsed dendritic cells: an approach for cancer immunotherapy. Vaccine 31, 1141–1156, doi: 10.1016/j.vaccine.2012.12.027 (2013). 23306369

[b6] Miles B. . Noncanonical dendritic cell differentiation and survival driven by a bacteremic pathogen. Journal of leukocyte biology 94, 281–289, doi: 10.1189/jlb.0213108 (2013). 23729500 PMC3714568

[b7] Ziegler-Heitbrock L. . Nomenclature of monocytes and dendritic cells in blood. Blood 116, e74–80, doi: 10.1182/blood-2010-02-258558 (2010). 20628149

[b8] Carrion J. . Microbial carriage state of peripheral blood dendritic cells (DCs) in chronic periodontitis influences DC differentiation, atherogenic potential. Journal of immunology (Baltimore, Md.: 1950) 189, 3178–3187, doi: 10.4049/jimmunol.1201053 (2012). PMC3459682 22891282

[b9] El-Awady A. R. . Porphyromonas gingivalis evasion of autophagy and intracellular killing by human myeloid dendritic cells involves DC-SIGN-TLR2 crosstalk. PLoS pathogens 10, e1004647, doi: 10.1371/journal.ppat.1004647 (2015). 25679217 PMC4352937

[b10] Strasser A., O’Connor L. & Dixit V. M. Apoptosis signaling. Annual review of biochemistry 69, 217–245, doi: 10.1146/annurev.biochem.69.1.217 (2000). 10966458

[b11] Hou W. S. & Van Parijs L. A Bcl-2-dependent molecular timer regulates the lifespan and immunogenicity of dendritic cells. Nat Immunol 5, 583–589 (2004). 15133508 10.1038/ni1071

[b12] Downward J. PI 3-kinase, Akt and cell survival. Seminars in cell & developmental biology 15, 177–182 (2004). 15209377 10.1016/j.semcdb.2004.01.002

[b13] Zeituni A. E., McCaig W., Scisci E., Thanassi D. G. & Cutler C. W. The native 67-kilodalton minor fimbria of Porphyromonas gingivalis is a novel glycoprotein with DC-SIGN-targeting motifs. Journal of bacteriology 192, 4103–4110, doi: 10.1128/jb.00275-10 (2010). 20562309 PMC2916435

[b14] Geijtenbeek T. B. . Mycobacteria target DC-SIGN to suppress dendritic cell function. The Journal of experimental medicine 197, 7–17 (2003). 12515809 10.1084/jem.20021229 PMC2193797

[b15] Gringhuis S. I., den Dunnen J., Litjens M., van der Vlist M. & Geijtenbeek T. B. Carbohydrate-specific signaling through the DC-SIGN signalosome tailors immunity to Mycobacterium tuberculosis, HIV-1 and Helicobacter pylori. Nat Immunol 10, 1081–1088, doi: 10.1038/ni.1778 (2009). 19718030

[b16] Caparros E. . DC-SIGN ligation on dendritic cells results in ERK and PI3K activation and modulates cytokine production. Blood 107, 3950–3958, doi: 10.1182/blood-2005-03-1252 (2006). 16434485

[b17] Birkenkamp K. U. & Coffer P. J. FOXO transcription factors as regulators of immune homeostasis: molecules to die for? Journal of immunology (Baltimore, Md.: 1950) 171, 1623–1629 (2003). 10.4049/jimmunol.171.4.1623 12902457

[b18] Burgering B. M. & Kops G. J. Cell cycle and death control: long live Forkheads. Trends in biochemical sciences 27, 352–360 (2002). 12114024 10.1016/s0968-0004(02)02113-8

[b19] Riol-Blanco L. . Immunological synapse formation inhibits, via NF-kappaB and FOXO1, the apoptosis of dendritic cells. Nature immunology 10, 753–760, doi: 10.1038/ni.1750 (2009). 19503105

[b20] Cao D., van Vollenhoven R., Klareskog L., Trollmo C. & Malmstrom V. CD25brightCD4 + regulatory T cells are enriched in inflamed joints of patients with chronic rheumatic disease. Arthritis research & therapy 6, R335–346, doi: 10.1186/ar1192 (2004). 15225369 PMC464877

[b21] Soilleux E. J. . DC-SIGN association with the Th2 environment of lepromatous lesions: cause or effect? The Journal of pathology 209, 182–189, doi: 10.1002/path.1972 (2006). 16583355

[b22] Sugiyama H. . Dysfunctional blood and target tissue CD4 + CD25high regulatory T cells in psoriasis: mechanism underlying unrestrained pathogenic effector T cell proliferation. Journal of immunology (Baltimore, Md.: 1950) 174, 164–173 (2005). 10.4049/jimmunol.174.1.164 PMC2903964 15611238

[b23] Zeituni A. E., Jotwani R., Carrion J. & Cutler C. W. Targeting of DC-SIGN on human dendritic cells by minor fimbriated Porphyromonas gingivalis strains elicits a distinct effector T cell response. Journal of immunology (Baltimore, Md.: 1950) 183, 5694–5704, doi: 10.4049/jimmunol.0901030 (2009). PMC2770168 19828628

[b24] Chung D. J. . Indoleamine 2,3-dioxygenase-expressing mature human monocyte-derived dendritic cells expand potent autologous regulatory T cells. Blood 114, 555–563, doi: 10.1182/blood-2008-11-191197 (2009). 19465693 PMC2713474

[b25] Munn D. H. & Mellor A. L. Indoleamine 2,3-dioxygenase and tumor-induced tolerance. Journal of Clinical Investigation 117, 1147–1154, doi: 10.1172/jci31178 (2007). 17476344 PMC1857253

[b26] Jauregui C. E. . Suppression of T-Cell Chemokines by Porphyromonas gingivalis. Infection and immunity 81, 2288–2295, doi: 10.1128/iai.00264-13 (2013). 23589576 PMC3697598

[b27] Hajishengallis G. & Lamont R. J. Breaking bad: Manipulation of the host response by Porphyromonas gingivalis. European journal of immunology 44, 328–338, doi: 10.1002/eji.201344202 (2014). 24338806 PMC3925422

[b28] van Rooijen N. & Hendrikx E. Liposomes for specific depletion of macrophages from organs and tissues. Methods in molecular biology (Clifton, N.J.) 605, 189–203, doi: 10.1007/978-1-60327-360-2_13 (2010). 20072882

[b29] van Rooijen N. & van Kesteren-Hendrikx E. “*In vivo*” depletion of macrophages by liposome-mediated “suicide”. Methods in enzymology 373, 3–16 (2003). 14714393 10.1016/s0076-6879(03)73001-8

[b30] Arnold L. . Analysis of innate defences against Plasmodium falciparum in immunodeficient mice. Malaria journal 9, 197, doi: 10.1186/1475-2875-9-197 (2010). 20618960 PMC2914061

[b31] Badell E. . Human malaria in immunocompromised mice: an *in vivo* model to study defense mechanisms against Plasmodium falciparum. The Journal of experimental medicine 192, 1653–1660 (2000). 11104807 10.1084/jem.192.11.1653 PMC2193098

[b32] Vaughan A. M., Kappe S. H. I., Ploss A. & Mikolajczak S. A. Development of humanized mouse models to study human malaria parasite infection. Future microbiology 7, 10.2217/fmb.2212.2227, doi: 10.2217/fmb.12.27 (2012). PMC3848604 22568719

[b33] Moreno A., Badell E., Van Rooijen N. & Druilhe P. Human malaria in immunocompromised mice: new *in vivo* model for chemotherapy studies. Antimicrobial agents and chemotherapy 45, 1847–1853, doi: 10.1128/aac.45.6.1847-1853.2001 (2001). 11353636 PMC90556

[b34] Boisvert J., Edmondson S. & Krummel M. F. Immunological synapse formation licenses CD40-CD40L accumulations at T-APC contact sites. Journal of immunology (Baltimore, Md.: 1950) 173, 3647–3652 (2004). 10.4049/jimmunol.173.6.3647 15356109

[b35] Kim K. D., Choe Y. K., Choe I. S. & Lim J. S. Inhibition of glucocorticoid-mediated, caspase-independent dendritic cell death by CD40 activation. Journal of leukocyte biology 69, 426–434 (2001). 11261790

[b36] Hanks B. A. . Re-engineered CD40 receptor enables potent pharmacological activation of dendritic-cell cancer vaccines *in vivo*. Nature medicine 11, 130–137, doi: 10.1038/nm1183 (2005). 15665830

[b37] Chen J. . A unique pattern of up- and down-regulation of chemokine receptor CXCR3 on inflammation-inducing Th1 cells. European journal of immunology 34, 2885–2894, doi: 10.1002/eji.200425318 (2004). 15368305

[b38] Pernis A. . Lack of interferon gamma receptor beta chain and the prevention of interferon gamma signaling in TH1 cells. Science 269, 245–247 (1995). 7618088 10.1126/science.7618088

[b39] Lloyd C. M. . CC chemokine receptor (CCR)3/eotaxin is followed by CCR4/monocyte-derived chemokine in mediating pulmonary T helper lymphocyte type 2 recruitment after serial antigen challenge *in vivo*. The Journal of experimental medicine 191, 265–274 (2000). 10637271 10.1084/jem.191.2.265 PMC2195756

[b40] Kaplan M. H., Schindler U., Smiley S. T. & Grusby M. J. Stat6 is required for mediating responses to IL-4 and for development of Th2 cells. Immunity 4, 313–319 (1996). 8624821 10.1016/s1074-7613(00)80439-2

[b41] Takahashi T. . Immunologic self-tolerance maintained by CD25(+)CD4(+) regulatory T cells constitutively expressing cytotoxic T lymphocyte-associated antigen 4. The Journal of experimental medicine 192, 303–310 (2000). 10899917 10.1084/jem.192.2.303 PMC2193248

[b42] Deaglio S. . Adenosine generation catalyzed by CD39 and CD73 expressed on regulatory T cells mediates immune suppression. The Journal of experimental medicine 204, 1257–1265, doi: 10.1084/jem.20062512 (2007). 17502665 PMC2118603

[b43] Gomez-Cabanas L. . Detecting apoptosis of leukocytes in mouse lymph nodes. Nature protocols 9, 1102–1112, doi: 10.1038/nprot.2014.078 (2014). 24743418

[b44] Naik S. H. . Development of plasmacytoid and conventional dendritic cell subtypes from single precursor cells derived *in vitro* and *in vivo*. Nature immunology 8, 1217–1226, doi: 10.1038/ni1522 (2007). 17922015

[b45] Gillis C., Haegerstrand A., Ragnarson B. & Bengtsson L. Rapid visualization of viable and nonviable endothelium on cardiovascular prosthetic surfaces by means of fluorescent dyes. J Thorac Cardiovasc Surg 108, 1043–1048 (1994). 7527111

[b46] Druilhe P. . A Malaria Vaccine That Elicits in Humans Antibodies Able to Kill Plasmodium falciparum. PLoS medicine 2, e344, doi: 10.1371/journal.pmed.0020344 (2005). 16262450 PMC1277929

[b47] Koo G. C., Hasan A. & O’Reilly R. J. Use of humanized severe combined immunodeficient mice for human vaccine development. Expert review of vaccines 8, 113–120, doi: 10.1586/14760584.8.1.113 (2009). 19093778 PMC2677709

[b48] Ito M. . NOD/SCID/gamma(c)(null) mouse: an excellent recipient mouse model for engraftment of human cells. Blood 100, 3175–3182, doi: 10.1182/blood-2001-12-0207 (2002). 12384415

[b49] Yacoub-Youssef H. . Engraftment of human T, B and NK cells in CB.17 SCID/beige mice by transfer of human spleen cells. Transplant immunology 15, 157–164, doi: 10.1016/j.trim.2005.07.002 (2005). 16412960

[b50] Lopez A. F., Strath M. & Sanderson C. J. Differentiation antigens on mouse eosinophils and neutrophils identified by monoclonal antibodies. British journal of haematology 57, 489–494 (1984). 6743568 10.1111/j.1365-2141.1984.tb02923.x

[b51] Hu Z., Van Rooijen N. & Yang Y. G. Macrophages prevent human red blood cell reconstitution in immunodeficient mice. Blood 118, 5938–5946, doi: 10.1182/blood-2010-11-321414 (2011). 21926352 PMC3228505

[b52] Arnold L. . Further improvements of the P. falciparum humanized mouse model. PLoS ONE 6, e18045, doi: 10.1371/journal.pone.0018045 (2011). 21483851 PMC3069031

[b53] Sela U., Olds P., Park A., Schlesinger S. J. & Steinman R. M. Dendritic cells induce antigen-specific regulatory T cells that prevent graft versus host disease and persist in mice. The Journal of experimental medicine 208, 2489–2496, doi: 10.1084/jem.20110466 (2011). 22084406 PMC3256961

[b54] Wathelet N. & Moser M. Role of dendritic cells in the regulation of antitumor immunity. Oncoimmunology 2, e23973, doi: 10.4161/onci.23973 (2013). 23734333 PMC3654603

[b55] Sallusto F. & Lanzavecchia A. Efficient presentation of soluble antigen by cultured human dendritic cells is maintained by granulocyte/macrophage colony-stimulating factor plus interleukin 4 and downregulated by tumor necrosis factor alpha. The Journal of experimental medicine 179, 1109–1118 (1994). 8145033 10.1084/jem.179.4.1109 PMC2191432

[b56] Wang Y. . Transforming growth factor beta-activated kinase 1 (TAK1)-dependent checkpoint in the survival of dendritic cells promotes immune homeostasis and function. Proceedings of the National Academy of Sciences of the United States of America 109, E343–352, doi: 10.1073/pnas.1115635109 (2012). 22308391 PMC3277515

[b57] Yang Y. . Acetylation of FoxO1 activates Bim expression to induce apoptosis in response to histone deacetylase inhibitor depsipeptide treatment. Neoplasia (New York, N.Y.) 11, 313–324 (2009). 10.1593/neo.81358 PMC2657887 19308286

[b58] Mellor A. L. & Munn D. H. IDO expression by dendritic cells: tolerance and tryptophan catabolism. Nature reviews. Immunology 4, 762–774, doi: 10.1038/nri1457 (2004). 15459668

[b59] Friedline R. H. . CD4+ regulatory T cells require CTLA-4 for the maintenance of systemic tolerance. The Journal of experimental medicine 206, 421–434, doi: 10.1084/jem.20081811 (2009). 19188497 PMC2646578

[b60] Kerdiles Y. M. . Foxo transcription factors control regulatory T cell development and function. Immunity 33, 890–904, doi: 10.1016/j.immuni.2010.12.002 (2010). 21167754 PMC3034255

[b61] Rodriguez-Fernandez J. L. . Rho and Rho-associated kinase modulate the tyrosine kinase PYK2 in T-cells through regulation of the activity of the integrin LFA-1. The Journal of biological chemistry 276, 40518–40527, doi: 10.1074/jbc.M102896200 (2001). 11489881

[b62] Lages C. S. . Functional regulatory T cells accumulate in aged hosts and promote chronic infectious disease reactivation. Journal of immunology (Baltimore, Md.: 1950) 181, 1835–1848 (2008). 10.4049/jimmunol.181.3.1835 PMC2587319 18641321

[b63] Ouyang W. . Foxo proteins cooperatively control the differentiation of Foxp3+ regulatory T cells. Nat Immunol 11, 618–627, doi: 10.1038/ni.1884 (2010). 20467422

[b64] Harada Y. . Transcription factors Foxo3a and Foxo1 couple the E3 ligase Cbl-b to the induction of Foxp3 expression in induced regulatory T cells. The Journal of experimental medicine 207, 1381–1391, doi: 10.1084/jem.20100004 (2010). 20439537 PMC2901074

[b65] Kuboniwa M. . Distinct roles of long/short fimbriae and gingipains in homotypic biofilm development by Porphyromonas gingivalis. BMC Microbiol 9, 105, doi: 10.1186/1471-2180-9-105 (2009). 19470157 PMC2697998

[b66] Njoroge T., Genco R. J., Sojar H. T., Hamada N. & Genco C. A. A role for fimbriae in Porphyromonas gingivalis invasion of oral epithelial cells. Infection and immunity 65, 1980–1984 (1997). 9125593 10.1128/iai.65.5.1980-1984.1997 PMC175257

[b67] Cutler C. W., Kalmar J. R. & Arnold R. R. Phagocytosis of virulent Porphyromonas gingivalis by human polymorphonuclear leukocytes requires specific immunoglobulin G. Infection and immunity 59, 2097–2104 (1991). 2037370 10.1128/iai.59.6.2097-2104.1991 PMC257971

[b68] Tan K. H. . Porphyromonas gingivalis and Treponema denticola exhibit metabolic symbioses. PLoS pathogens 10, e1003955, doi: 10.1371/journal.ppat.1003955 (2014). 24603978 PMC3946380

[b69] Bentley C. . Influence of chylomicron remnants on human monocyte activation *in vitro*. Nutrition, metabolism, and cardiovascular diseases: NMCD 21, 871–878, doi: 10.1016/j.numecd.2010.02.019 (2011). PMC3212651 20674313

[b70] Robinson M. J., Sancho D., Slack E. C. & LeibundGut-Landmann S. & Reis e Sousa, C. Myeloid C-type lectins in innate immunity. Nat Immunol 7, 1258–1265, doi: 10.1038/ni1417 (2006). 17110942

