# Supplementary material for: Human IDO-competent, long-lived immunoregulatory dendritic cells induced by intracellular pathogen, and their fate in humanized mice
Source: Sci Rep. 2017 Feb 15;7:41083. doi: 10.1038/srep41083 (PMC5309771; doi:10.1038/srep41083)
Supplement: Supporting Information [file srep41083-s1.pdf]

Human IDO-competent, long-lived immunoregulatory dendritic cells induced by intracellular pathogen, and their fate in humanized mice

Rajeev K. Tyagi[1,3*], Brodie Miles[2*], Rajesh Parmar[3], Neeraj K. Garg[4], Sarat K. Dalai[3], Babak Baban[5] & Christopher W. Cutler[1*]

[1]Department of Periodontics, College of Dental Medicine, Georgia Regents University, Augusta, GA 30912, USA

[2]Division of Infectious Diseases, Anschutz Medical Campus, University of Colorado Denver, Aurora, CO 80045, USA

[3]Institute of Science, Nirma University, Sarkhej-Gandhinagar Highway, Ahmedabad 382481, Gujarat, India

[4]Drug Delivery Research Group, University Institute of Pharmaceutical Sciences, UGC center of Advanced Studies, Panjab University, Chandigarh, India

[5]Department of Oral Biology, Georgia Regents University, Augusta, GA 30912

*Contributed equally to the present paper

Corresponding author: R.K.T. (rajeev.gru@gmail.com) and C.W.C. (chcutler@augusta.edu)

Condensed title: Long-lived PDDCs & their tracking in humice

Supporting Information-Figure with captions

SI Figure S1A:

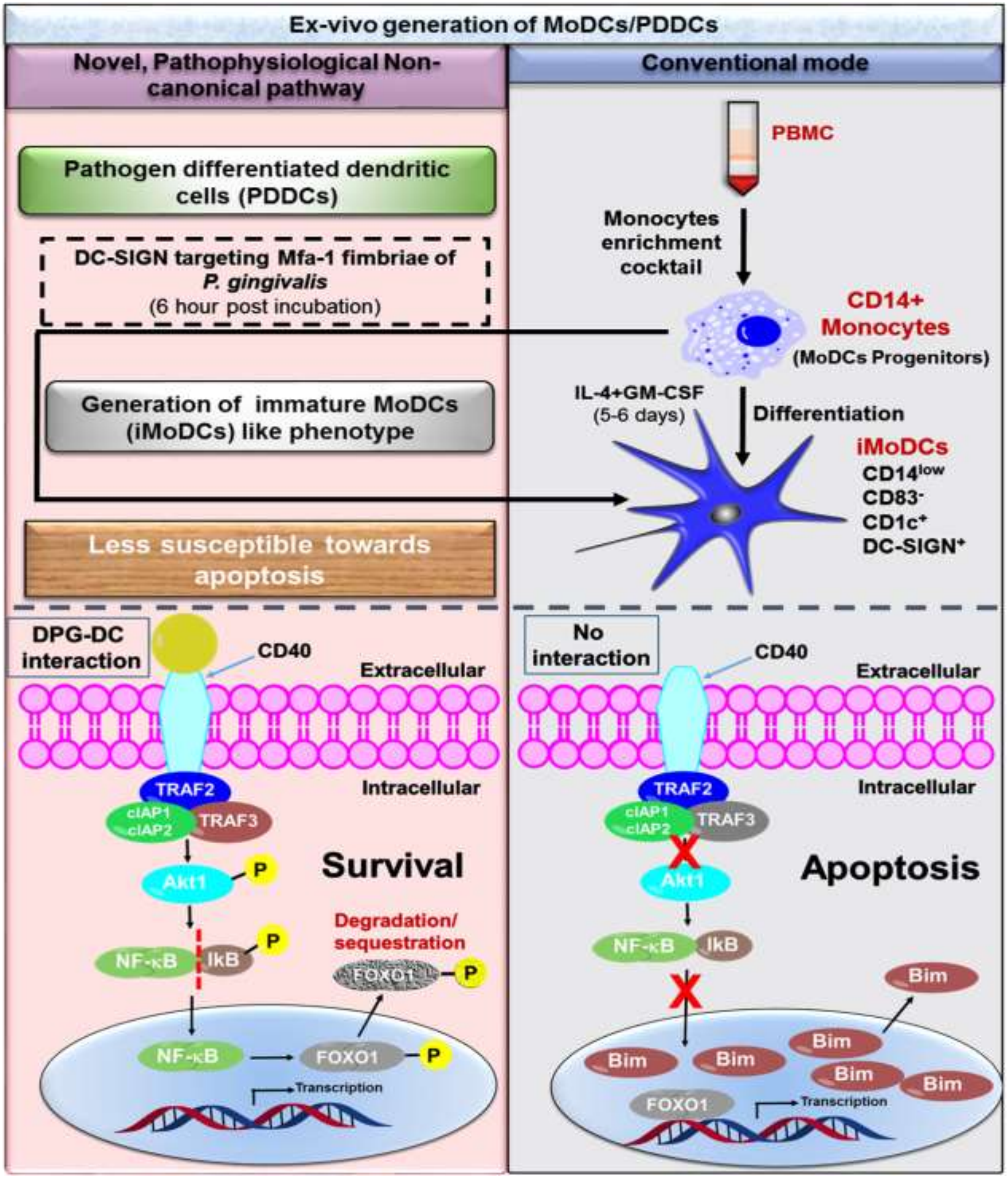

SI Figure S1B:

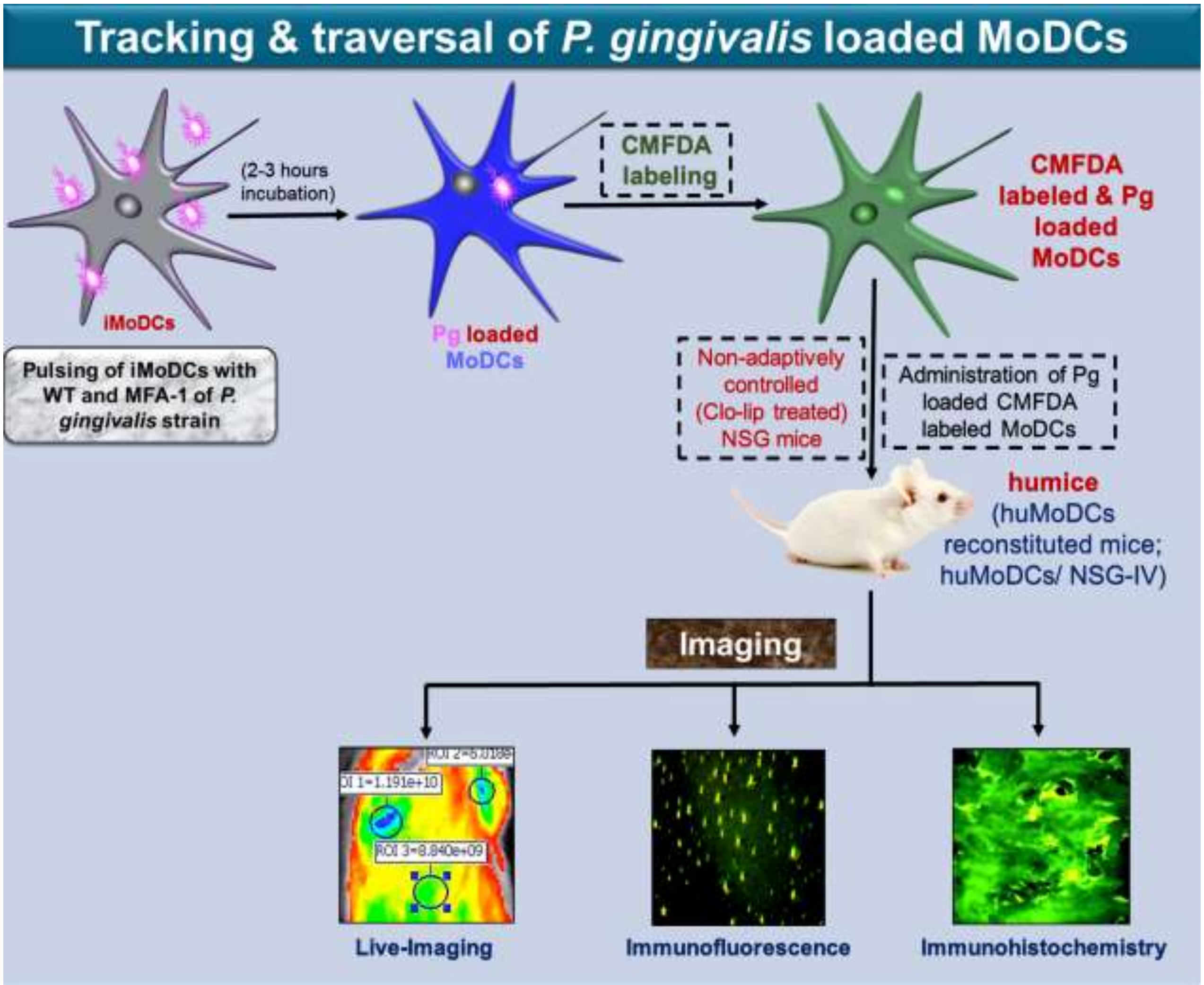

SI Figure S1: 1A) *Ex-vivo* generation of conventional monocyte derived dendritic cells through differentiation of CD14$^+$ monocytes into dendritic cell phenotype. The novel, pathophysiological, non-canonical pathway of differentiation of monocytes into immature DCs which are less susceptible to become apoptotic as compared to MoDCs, **1B)** Tracking and traversal of *P. gingivalis* loaded MoDCs in reconstituted humanized mice showing long-lasting survival and thereby residence in deep-seated tissues.

SI Figure S2:

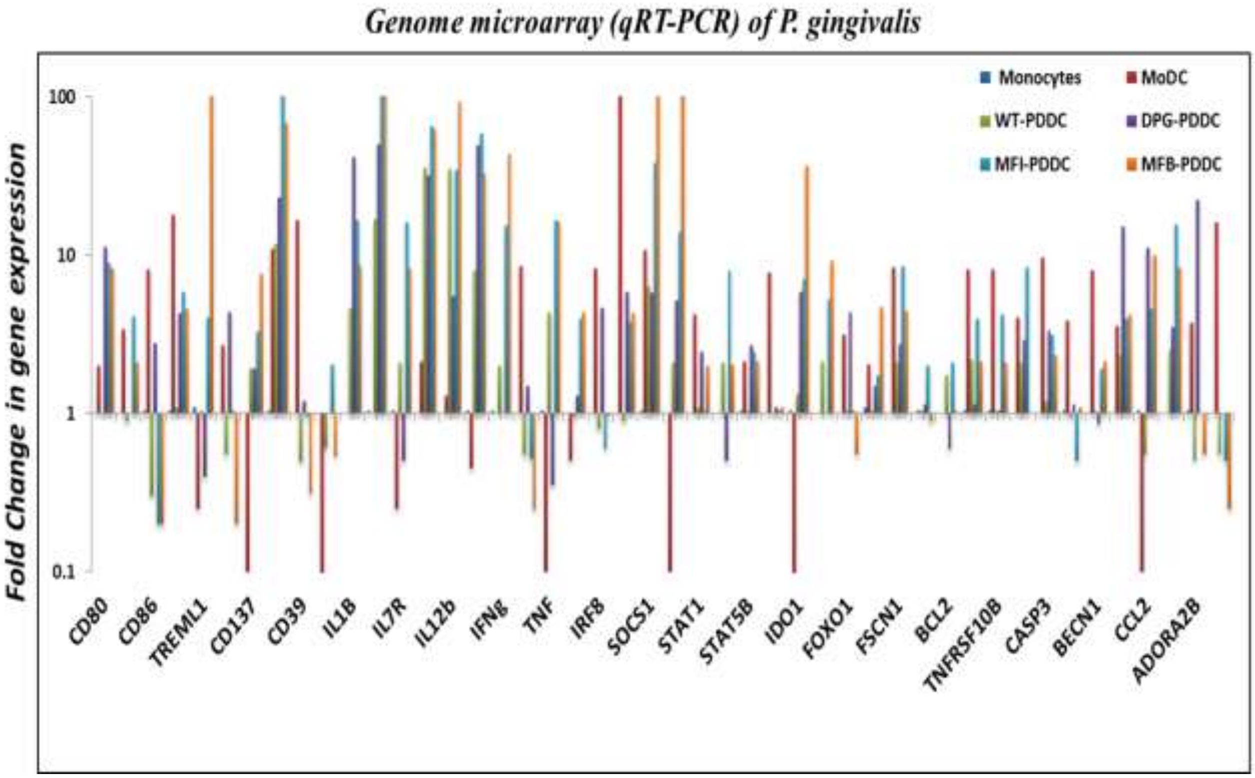

SI Figure S2: Genome microarray of *P. gingivalis* of monocyte-derived DCs (MoDCs) and pathogen differentiated dendritic cells (PDDCs) regulate expression of various markers relative to monocytes. Monocytes were isolated using human monocyte enrichment cocktail (RosetteSep), and were cultured in presence of growth factors (GM-CSF and IL-4) for 6 days to generated MoDCs, and 200K-400K monocytes/well distributed in six-well plate, and infected immediately with wild type, minor (mfa-1) fimbriae, major (MFI) fimbriae and double (MFB) isogenic mutant(s) of *Porphyromonas gingivalis* at 1 MOI. MoDCs and PDDCs were confirmed for their immature DC phenotype ($CD14^{low}CD83^{-}CD1c^{+}DC^{-}SIGN^{+}$) on day 6, and 6 hrs post-infection respectively. All markers were designed in triplicates on array plates. The fold change (in gene expression) value greater than one indicates a positive value, or an up-regulation of gene expression, and less than one indicates a negative value, or down-regulation of gene expression.

SI Figure S3:

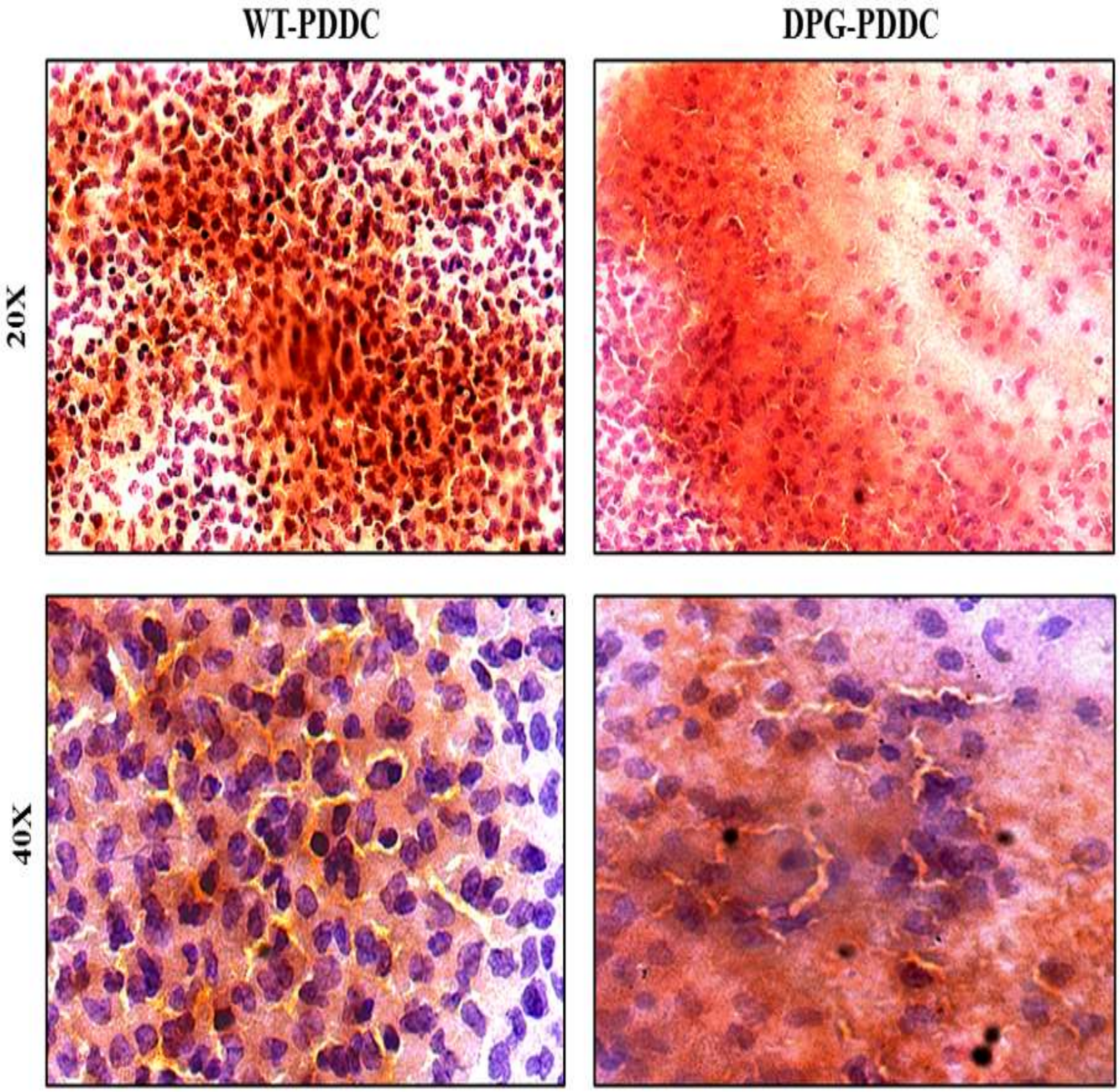

SI Figure S3: MoDCs show higher rate of apoptosis than PDDCs. The expression of FOXO1 was observed on PDDCs (WT and DPG) by immunocytochemistry performed on cytospin of PDDCs using FOXO1 specific monoclonal antibody.

SI Figure S4:

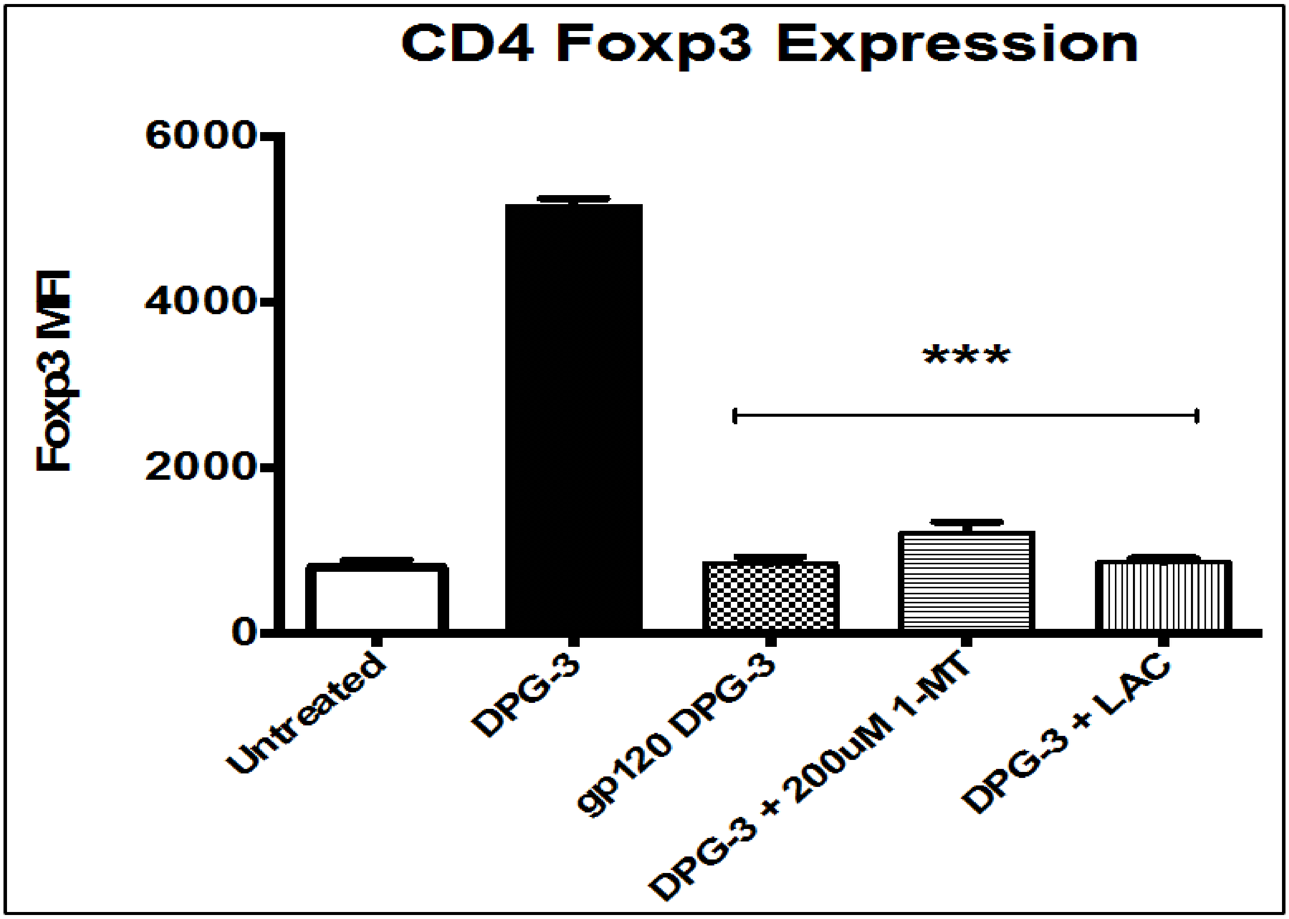

SI Figure S4: DPG-PDDCs and DPG-DCs and CD4 co-cultures express Foxp3^{+} cells. DPG-3 PDDCs induce IDO-dependent regulatory T cell responses from naïve CD4^{+} T cells. PDDC groups were generated for 24 hours and then cultured with autologous CD4^{+} T cells for 5 days. The Foxp3 expression was significantly increased in DPG-3 PDDC co-culture compared to MoDC controls. Foxp3 expression was significantly decreased when DPG-3 uptake was inhibited with HIV gp120 or cells were cultured in the presence of the IDO inhibitor 1-MT or leukocyte activation cocktail [67].

SI Figure S5:

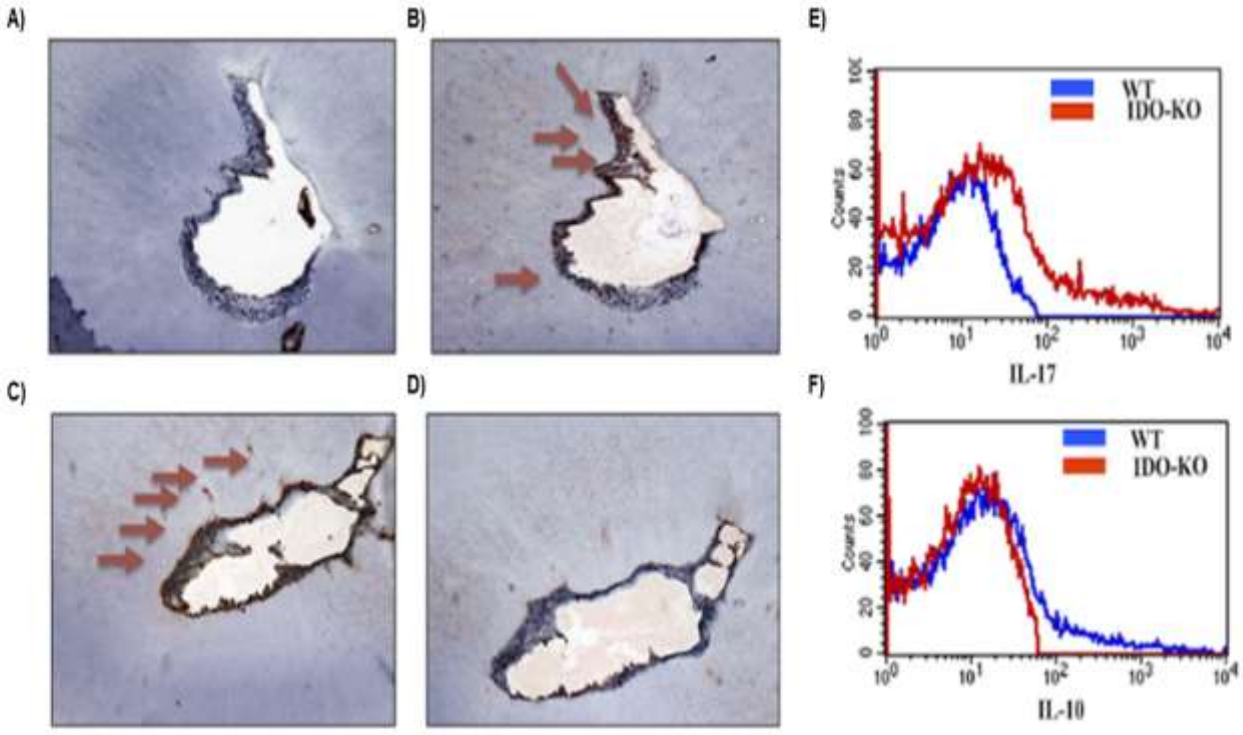

SI Figure S5: IDO plays a determinant role in the profile of cytokine expression in endotoxin-induced gingivitis. **A)** WT (IDO sufficient) mice showed a minimum level of IL-17 (pro-inflammatory) expression, while **B)** IDO-KO (IDO deficient) mice showed markedly increased level of IL-17 expression in gingival tissue (red arrows). **C)** WT (IDO sufficient) mice showed markedly higher level of level of IL-10 (anti-inflammatory) expression (red arrows) compared with IDO-KO (IDO deficient) mice **D)** that showed minimal expression level of IL-10 expression in gingival tissue (200X magnification). Consistent with IHC analysis, flow cytometric analysis showed higher level of IL-17 expression **E)** and lower level of IL-10 expression (F) in IDO-KO mice compared to their WT (IDO sufficient) counterparts.

SI Figure S6:

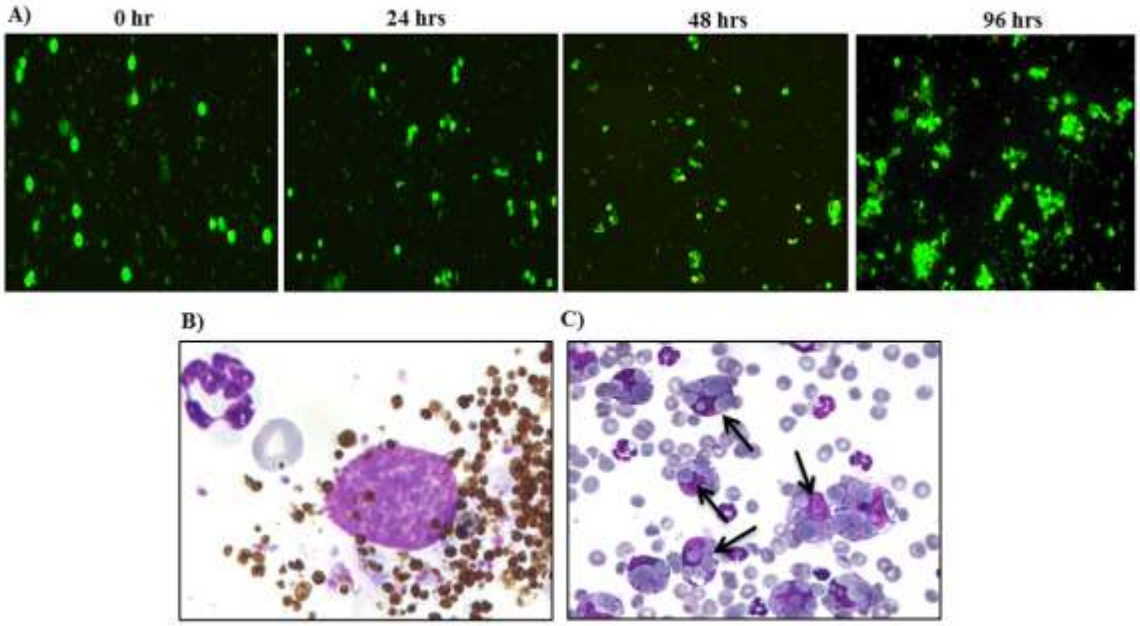

SI Figure S6: Sustainability of signals recorded from human monocytes labeled with CellTracker green CMFDA dye. **A)** Monocytes were isolated using human monocyte enrichment cocktail (RosetteSep). The monocytes were stained with CMFDA (10µM) following manufacturer's recommendations. 200K cells were cytospine onto slides and fixed with 4% paraformaldehyde, and were visualized under fluorescence microscope. The labeled MN showed signals for 96 hr. The present results were shown consistency in two independent experiments. The myeloablation in NSG mice to further reduce their residual immunity, **B)** monocyte and PMN appears inactive with pigments, **C)** phagocytosis of RBCs by active macrophages. Cells from monocyte-macrophage lineage in immunodeficient (NSG) mice are very active and clear human cells from mouse's periphery. The clodronate-loaded liposome is needed to control number of active macrophages to achieving substantial human cell engraftment. Microphotographs were taken from blood smears drawn from mice that received *P. gingivalis* infection through intravenous route, and stained with geimsa.

SI Figure S7:

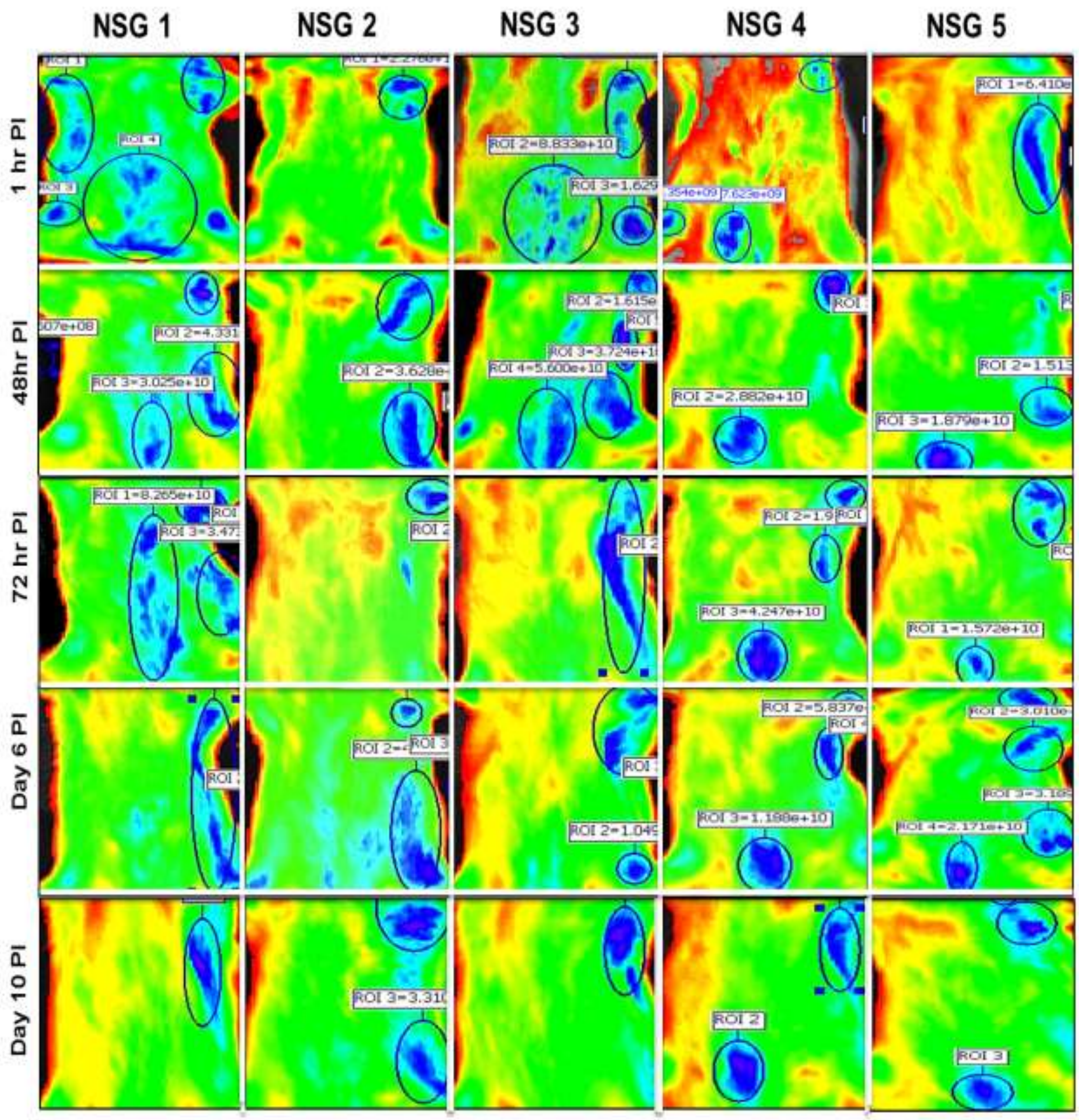

SI Figure S7: Tracking of CMFDA labeled human monocytes in controlled humanized mice. Monocytes were isolated using human monocyte enrichment cocktail (RosetteSep, StemCells technology) following manufacturer's instructions. NSG mice were intraperitoneally injected three times with clodronate-loaded liposomes (100µl reconstituted in 300µl RPMI) at every 3

days interval prior to CMFDA (15μM) labeled human $CD14^+$ monocytes administration. Each mouse received 4.5 million CMFDA-MN through intravenous route. The persistence of green signals by labeled MN until day 10 post-injection. The present results were shown consistency in two independent experiments.

SI Figure S8:

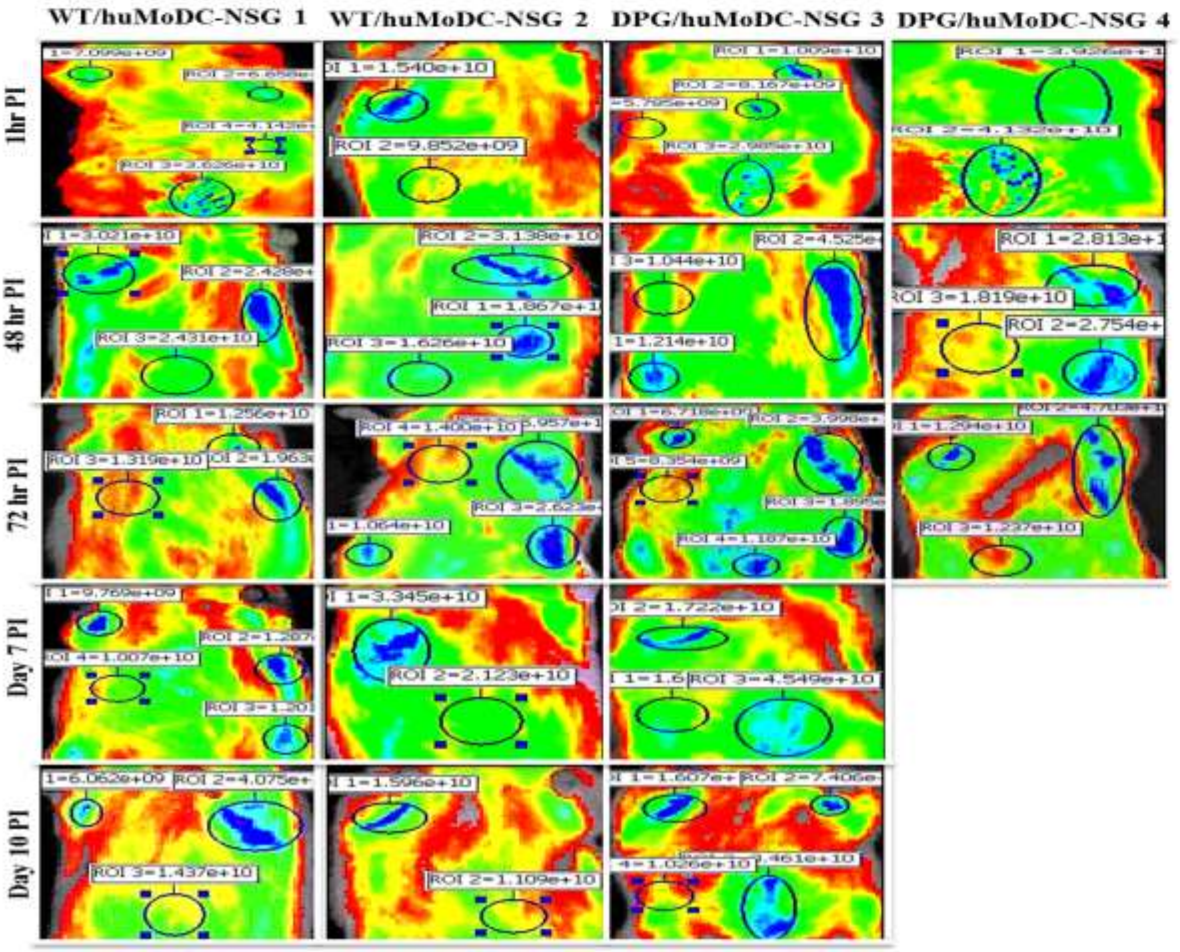

SI Figure S8: Tracking green CMFDA labeled monocyte-derived DCs in controlled humanized mice. Monocytes were isolated using human monocyte enrichment cocktail (RosetteSep), and were cultured in the presence of growth factors (GM-CSF and IL-4) for 6 days. MoDCs were confirmed for their immature DC phenotype ($CD14^{low}CD83^{-}CD1c^{+}DC\text{-}SIGN^{+}$) on day 6. The phenotypically characterized MoDCs were infected with wild type and minor fimbriated (DPG) strains of *Porphyromonas gingivalis* at 1 MOI. NSG mice received three injections of clodronate-loaded liposomes (100µl in 300µl RPMI) at every 3 day interval through intraperitoneal route prior to receiving infected MoDCs labeled with cell tracker CMFDA

(15µM). NSG 1 and 2 received 3.8 million labeled and WT pulsed MoDC and NSG 3 and 4 received 3.8 million labeled and DPG pulsed MoDC through intravenous route. The persistence of green signals by labeled MoDCs was recorded until day 10 post injection. The present results were shown consistency in two independent experiments.

SI Figure S9:

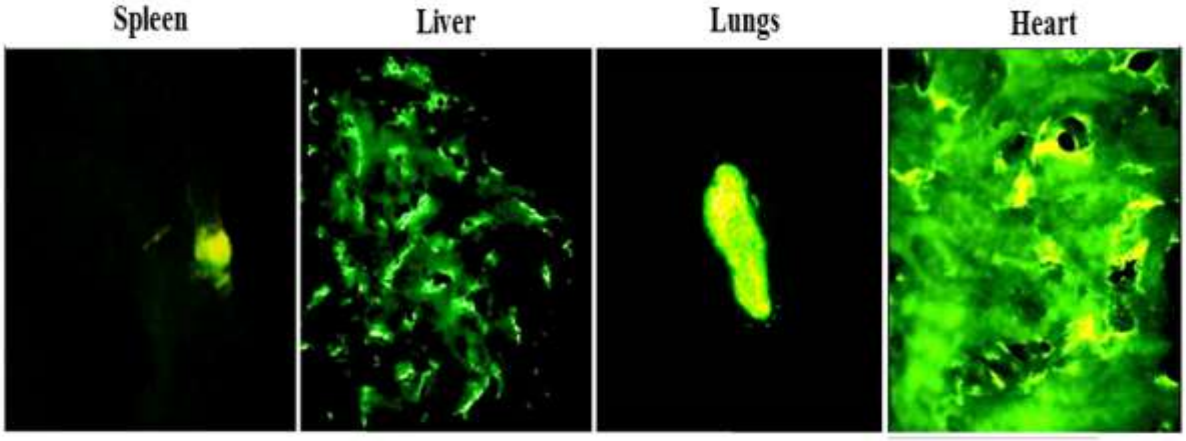

SI Figure S9: Microscopic analysis of fixed tissue sections of wild type *P. gingivalis* loaded and CMFDA labeled MoDCs. Mice were euthanized on day 4 post-injection of WT/huMoDC-NSG and different organs; spleen, liver, lungs, and heart were collected and cryopreserved in liquid N_2. The frozen tissue were then processed for cryo-sectionning by cryostat. Sections were fixed with 4% paraformaldehyde and then visualized under microscope in bright-field light. These results were reproducible in 2 NSG mice reconstituted with infected MoDCs.
